# Integrated charge excitation triboelectric nanogenerator

**DOI:** 10.1038/s41467-019-09464-8

**Published:** 2019-03-29

**Authors:** Wenlin Liu, Zhao Wang, Gao Wang, Guanlin Liu, Jie Chen, Xianjie Pu, Yi Xi, Xue Wang, Hengyu Guo, Chenguo Hu, Zhong Lin Wang

**Affiliations:** 1 Department of Applied Physics, State Key Laboratory of Power Transmission Equipment & System Security and New Technology, Chongqing University, 400044 Chongqing, P. R. China; 2 Beijing Institute of Nanoenergy and Nanosystems, Chinese Academy of Sciences, 100083 Beijing, P. R. China; 3 School of Materials Science and Engineering, Georgia Institute of Technology, Atlanta, GA 30332 USA

## Abstract

Performance of triboelectric nanogenerators is limited by low and unstable charge density on tribo-layers. An external-charge pumping method was recently developed and presents a promising and efficient strategy towards high-output triboelectric nanogenerators. However, integratibility and charge accumulation efficiency of the system is rather low. Inspired by the historical development of electromagnetic generators, here, we propose and realize a self-charge excitation triboelectric nanogenerator system towards high and stable output in analogy to the principle of traditional magnetic excitation generators. By rational design of the voltage-multiplying circuits, the completed external and self-charge excitation modes with stable and tailorable output over 1.25 mC m^−2^ in contact-separation mode have been realized in ambient condition. The realization of the charge excitation system in this work may provide a promising strategy for achieving high-output triboelectric nanogenerators towards practical applications.

## Abstract (web summary)

Triboelectric nanogenerators may benefit Internet-of-Things era energy demands, but application is hindered by low charge density. Here the authors maximize charge density in ambient conditions and achieve stable power generation in a triboelectric nanogenerator that can realize external and self-excitation.

## Introduction

With rapid development of portable, wearable electronics, and the Internet-of-Things, great efforts have been devoted to developing sustainable, mobile and distributed power sources for the energy of a new era^1–5^. Meanwhile, ambient mechanical energy associated with human activities provides an ideal power source for energy harvesting. Compared with conventional electromagnetic generators^6^ (EMGs), the triboelectric nanogenerator^7^ (TENG) has merits of light weight, low cost, wide choice of materials, and effectiveness in low-frequency energy harvesting that have attracted great attention in recent years^8–19^. The triboelectric nanogenerator has also been considered as a technology that is complementary to the electromagnetic generator^8,20–22^. However, a critical issue of the TENG is the low charge density^23–25^, which is quadratic to the power output and largely limits its practical applications^12^.

**Fig. 1:**
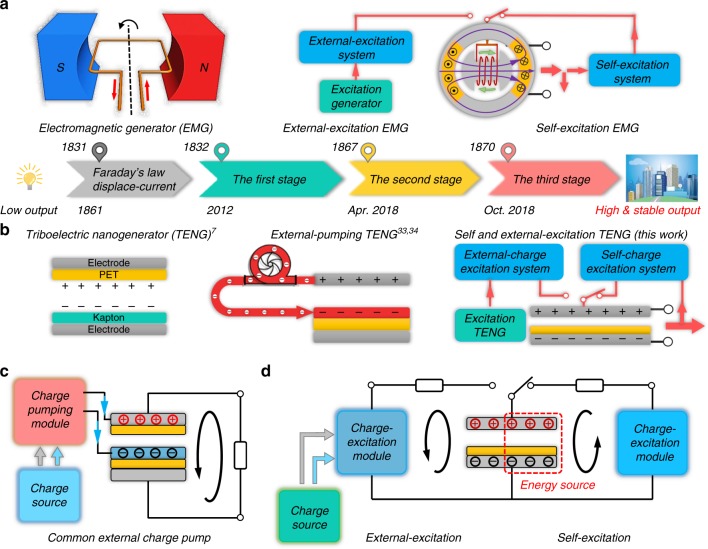
Historical development stages of mechanical energy converting device. **a** The development of electromagnetic generator (EMG) from faraday’s law to self-excitation EMG. **b** The development of triboelectric nanogenerator (TENG) from Maxwell displacement current to self-excitation TENG. **c** The fundamental system scheme of traditional external charge pump methods for improving the output density of TENG. **d** The fundamental scheme of both external and self-charge excitation TENG proposed in this work

In order to improve the charge density, much research has been focused on materials selection^26^, surface modification^27^, contact improvement^28^, and so on^29–34^, which can, to some extent, increase the charge density to hundreds of μC m^−2^. By studying Paschen’s law in a TENG model^29^, the charge density has reached 1.003 mC m^−2^ level for the first time in high vacuum environment^32^. Very recently, an external charge pump method has been reported, and the 1.02 mC m^−2^ output charge density has been realized in ambient conditions^34^, which may solve packaging issues in previous works. In referring to the developmental stages of electromagnetic generators (as shown in Fig. 1a), the fundamental principle of this method is similar to the external magnetic excitation generator^35^ (the second stage of EMG). Although, it is a great strategy to realize high and stable output, the system integration is a critical issue for this kind of external-excitation generator. Moreover, to quickly reach the saturated state and to consider the charge leakage of the system, a larger external-excitation device is needed. Therefore, inspired by the self-excitation EMG^6,36^ (the third stage of EMG), the development of a self-charge excitation TENG that utilizes part of the energy output from the TENG itself to enhance its working charge density is highly desired and also pushes the electricity generation of the TENG to the next stage (Fig. 1b).

Here, we develop a different working mechanism for a TENG system by charge transferring between the TENG capacitor and external capacitors. In such a working principle, we propose a strategy to excite charges directly on the electrodes of a TENG rather than on the dielectric tribo-layer or floating metal layer^30,34^. Utilizing voltage-multiplying circuits (VMC), we successfully realize both external charge excitation (ECE) and self-charge excitation (SCE) in a TENG system with the effective charge output density (ECD) up to 1.25 mC m^−2^ in ambient conditions when using a 5-μm dielectric Kapton film. In this work, the effects of many factors, such as the dielectric type, thickness, electrode materials, operation frequency, environmental humidity, etc., on the output charge density of our excitation TENG system are systematically studied. A comparison of recent charge excitation TENG works is also presented. An exponential charge accumulation property is obtained for the self-charge excitation TENG (SCE-TENG), which shows ultra-fast charge excitation efficiency (reaching saturation state within 50 s at 1 Hz). This work may provide a new platform for TENGs to achieve ultrahigh and stable power generation in a charge excitation TENG system for large-scale power applications.

## Results

### Fundamental concept of charge excitation nanogenerator

The basic concept of charge excitation TENG is similar to the magnetic excitation generator (Fig. 1a, b). It is to utilize either an external or self-excitation system to supply the working component of the main generator to produce a stronger and sustainable magnetic/electric field, thus generating a high and stable output power. Previous works^34^ demonstrated a kind of external charge pumping TENG system (Fig. 1c). The fundamental principle of the system is to create a floating charge layer in the main TENG (Supplementary Figure 1a) through a pump TENG. In this mode, charge source, charge pumping module and floating layer forms an independent system, and the main TENG and the output load is another independent system.

Different from the work above, we proposed another charge excitation strategy and working mode of the main TENG that can realize both external and self-excitation (Fig. 1d). In this system, the excited charge is supplied on the electrode of the main TENG, and, the charge excitation system, the main TENG and output load form an independent system. Owing to the capacitance characteristics of the charge excitation module, the output for the external load is realized by the charge transfer between the main TENG and ceramic capacitors in charge excitation module (Supplementary Figure 1b). Especially, with rational design of the charge excitation module, the charge stored in it can be boosted up to feed back the TENG itself during the discharging process, and thus achieve the self-excitation TENG. The detailed design and mechanism are presented in the following sections.

### Principle of external charge excitation nanogenerator

**Fig. 2:**
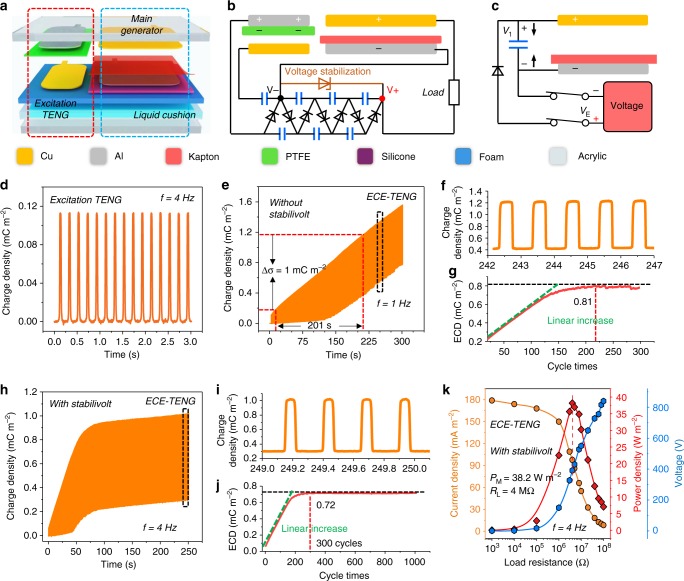
Mechanism and output of external charge excitation nanogenerator. **a** Structural illustration of ECE-TENG. **b** The systematical electric circuit of ECE-TENG. **c** Simplified working components of ECE-TENG. **d** The basic output charge of the excitation TENG under 4 Hz operation frequency. **e** The dynamic output charge accumulation process of ECE-TENG without voltage stabilization element under 1 Hz operation frequency. **f** The detailed output charge curve from the dashed area. **g** The effective charge density (ECD) versus operation cycles. **h** The dynamic output charge accumulation process of ECE-TENG with voltage stabilization element under 4 Hz operation frequency. **i** The detailed output charge curve from the dashed area. **j** The ECD versus operation cycles. **k** The current, voltage and power output of ECE-TENG with voltage stabilization under various external load (sinusoidal motion with 4 Hz frequency). The thickness of the dielectric Kapton film here is 9 μm. The effective charge output density is calculated from main TENG part

The 3D structural scheme of the external charge excitation TENG (ECE-TENG) is illustrated in Fig. 2a. It contains a basic excitation TENG and the main TENG both working in the contact-separation mode. In order to achieve a relatively large capacitance variation of the main TENG, 9-μm Kapton film was used as the dielectric layer. Moreover, flexible silicone, foam, and liquid cushion in the bottom were employed as buffer layer to ensure the effective contact between the electrode and dielectric film. The detailed device fabrication process is described in the methods section. Figure 2b shows the electric circuit loop of the whole ECE-TENG system, and Fig. 2c is the simplified electric components scheme. Similar to previous studies, the AC output from the external TENG was applied to the electric circuit to produce a DC output excitation voltage *V*_*E*_ and thus supply the main TENG. Differently, the charge was supplied on the electrode of the main TENG, and the AC output was realized by the charge transfer between the main TENG and ceramic capacitor (Supplementary Figure 1).

For the main TENG in our work, initially, charges (*σ*_M0_) would inject from the voltage source into the main TENG and build up an excitation voltage *V*_E_ when it is in contact state (maximum capacitance). When the two electrodes are separate, the voltage would increase due to the decrease of the capacitance of the main TENG. Consequently, charges would transfer from the main TENG to the charge storage capacitor *C*_S_ to reach an equilibrium state. In the following contact process, the charges would flow back to the main TENG and generate power. The charge density *σ*_M0_ and *V*_E_ can be described by equation (1).

1 $$\sigma _{\mathrm{M0}} = \frac{{\varepsilon _0\varepsilon _{\mathrm{r}}}}{d} \cdot V_{\mathrm{E}}$$

2 $$C_{\mathrm{S}} = \frac{1}{{\frac{1}{{C0}} + \frac{1}{{C1}} + \frac{1}{{C2}}}}$$

where *d* and *ε*_r_ are thickness and relative permittivity of the dielectric film, and *ε*_0_ is the vacuum dielectric constant. The detailed working mechanism and theoretical analysis of the main TENG are presented in Supplementary Note 1, 2 and Supplementary Figure 2.

From above analysis, a high and stable *V*_E_ would lead to a high and stable output. Therefore, in this work, a voltage-multiplying circuit (VMC) and Zener diode were used to boost and stabilize the voltage output from excitation TENG to a designed value (Supplementary Figure 3a). A photograph of VMC is depicted in Supplementary Figure 3b and Fig. 4a, which consists of seven rectifier diodes and seven ceramic capacitors. Here, the charge storage capacitor *C*_S_ can be described by equation (2). With an AC input *V*_0_, the DC output voltage can be boosted to 6 *V*_0_^37^. The mechanism of VMC for voltage boosting is presented in Supplementary Figure 3c and Supplementary Note 3.

It is worth noting that, according to the Paschen’s law^38,39^, when *V*_E_*/σ*_M0_ exceeds a critical value *V*_CE_*/σ*_C_, air breakdown between the surface of electrode and dielectric film would happen, which causes the decrease and instability on the output performance. After air breakdown, the dielectric film would be positively (oppositely) charged during corona discharge process (Supplementary Figure 5 and Supplementary Note 4). The experimental results in Supplementary Figure 6a and Supplementary Movie 1 prove the existence of opposite charges on the dielectric layer caused by air breakdown under strong electric field. As excessive voltage can cause dielectric film breakdown, so the use of Zener diode is not only to stabilize voltage but also to avoid dielectric film breakdown by releasing the surplus excited charges (Supplementary Figure 3d).

### Performance of external charge excitation nanogenerator

To measure the electric performance of ECE-TENG, a programmable liner motor was used to create the contact-separation movement. The charge density produced by the excitation TENG is 0.113 mC m^−2^ at 4 Hz (Fig. 2d), which is used to supply charges to the main TENG through the VMC. Without voltage stabilization, the charge density in the main TENG increases with operation time, while the baseline begins to shift up after charge density reaches a critical value *σ*_*C*_ as shown in Fig. 2e, f. The shifting of the charge output is caused by air breakdown. Correspondingly, the efficient charge density (ECD) that can output to drive external load linearly increases in the initial stage, and then decreases slowly after reaching the maximum of 0.81 mC m^−2^, as shown in Fig. 2g. To avoid the air breakdown, a suitable Zener diode (insert of Supplementary Figure 3a) is important for VMC to stabilize the voltage to a certain value. Obviously, the charge output (Fig. 2h, i and Supplementary Movie 2) with voltage stabilization is much more stable than that without voltage stabilization (Fig. 2e), from which the ECD shows a stable value of 0.72 mC m^−2^ (Fig. 2j), close to 0.75 mC m^−2^ obtained without voltage stabilization at 4 Hz (Supplementary Figure 7a). Similarly, the short-circuit current rises at first, and then fixes at a stable current of 201 mA m^−2^ with voltage stabilization (Supplementary Figure 7b). The voltage rapidly reaches a stable state with the maximum of 815 V, but the fluctuation is relatively larger compared to the current (Supplementary Figure 7c). The influence of frequency from 1 to 6 Hz on the electric performance is shown in Supplementary Figure 7a and d. The current approximates a linear increase, and voltage increases rapidly first and then slows down with an increase in frequency. The maximum current of 252 mA m^−2^ and voltage of 817 V are obtained at 6 Hz. The ECD decreases linearly from 0.81 mC m^−2^ to 0.71 mC m^−2^ with an increasing frequency, because of the charge/discharge time of capacitors. Figure 2k shows the output current and power density at different resistance from 1 KΩ to 100 MΩ, and the maximum power density reaches to 38.2 W m^−2^ with load of 4 MΩ at 4 Hz. It is worth noting that, when using a thinner dielectric Kapton film (5 μm), the output charge density would further increase to 1.26 mC m^−2^ as shown Table 1 and in Supplementary Figure 8a-d.

**Table 1:** The parameters and output properties of charge excitation TENG

Type	Excitation TENG	Main TENG	Thickness (μm)	Current (mA m^−2^)	ECD (mC m^−2^)	Charge accumulation time (s/mC m^−2^)
ECE-TENG	Al/PTFE/Cu	Cu/Kapton/Al	9	252	0.81	201
			5	426	1.26	218
SCE-TENG	—	Cu/Kapton/Al	9	233	0.83	32
			5	409	1.25	23

### Some critical factors on effective charge density

As the charge leakage is unavoidable in electronic components, the charges can be accumulated on the electrodes of the ECE-TENG only when the current supplied by excitation TENG is higher than the leakage current of all the components in the circuit. Supplementary Table 1 shows the leakage current of the main components used in this work. The excitation TENG with area of 5 cm^2^ can produce an average current of 120 nA at 1 Hz, which is far greater than the leakage current of about 25 nA (without Zener diode). Hence, the charge can be accumulated effectively at 1 Hz for the ECE-TENG.

The influence of dielectric layers and metal electrode materials on the main TENG of ECE-TENG were also investigated, as shown in Supplementary Note 5, which indicated that better performance is obtained in a thinner dielectric layer and Cu/Kapton-Al structure. The results confirm the Paschen’s law^29^ for the charge excitation TENG. Four different cushions (three foams and one c-cushion) are compared, indicating that the composite liquid cushion is the best one for the enhancement of ECD. The influence of humidity on the ECD is also measured for different dielectric materials, from which we know that the ECD decreases slowly before 50% RH and has the largest value at 5% RH. According to these investigations, we choose the 10 cm^2^ main TENG with Cu/Kapton-Al structure (Kapton thickness of 9 μm) and composite liquid cushion, measured at temperature of 293 K and humidity of 5% RH. The detailed discussion is presented in Supplementary Note 5 and Supplementary Figures 9–11.

### Principle of self-charge excitation nanogenerator

**Fig. 3:**
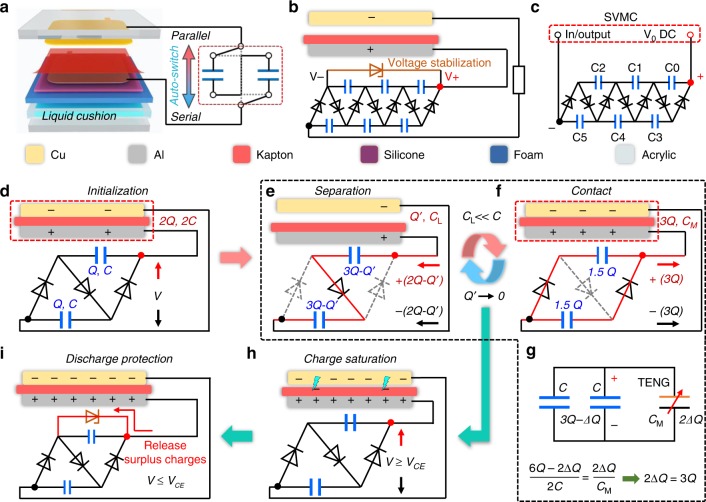
Principle of the self-charge excitation triboelectric nanogenerator. **a** The fundamental scheme of self-charge excitation triboelectric nanogenerator (SCE-TENG), the auto-switch can change capacitors from parallel to serial connection during the operation cycle. **b** The systematical electric circuit scheme of SCE-TENG. **c** The input/output node and scheme of self-voltage-multiplying circuit (SVMC). **d** The charge distribution of SCE-TENG in initial state (simplified from one SVMC unit). **e**–**g** Charge excitation process during periodically contact-separation cycle. **h**, **i** Air breakdown caused by high charge density and discharge protection circuit, respectively

Figure 3a shows the structural scheme of SCE-TENG system. The basic power generation mechanism of the main TENG is based on the charge transfer between two groups of capacitors, which is the same as ECE-TENG (Supplementary Note 1, 2 and Supplementary Figure 2). Differently, if the external capacitor group can realize the automatic switch from the parallel to serial connection during contact and separation process, the doubled charges from the parallel-connected capacitors could feed back to TENG component and thus implement the function of self-excitation. In this work, we use a self-voltage-multiplying circuit (SVMC) designed from VMC to build a SCE-TENG system. The detailed electric circuit scheme of the SCE-TENG and SVMC used in this work are depicted in Fig. 3b, c and Supplementary Figure 4b, respectively. From another perspective, the SCE-TENG can also be derived from the ECE-TENG system, Supplementary Figure 12 illustrates its evolution diagram from ECE-TENG.

To simplify the discussion, we chose one SVMC unit that consists of three rectifier diodes and two ceramic capacitors, to elaborate the fundamental self-charge excitation mechanism. In the initial state (Fig. 3d), we define the capacitance and charge quantity of TENG and ceramic capacitors are 2*C*, 2*Q* and *C*, *Q*, respectively. Correspondingly, there should be a voltage *V* between the two electrodes. When the two electrodes are separated, the capacitance of TENG would dramatically decrease to *C*_L_, and consequently, the voltage *V* would increase, leading to charge transfer 2*Q*–*Q’* from TENG to the ceramic capacitors to reach an equilibrium state, where *Q’* is real-time charge quantity of the main TENG. During this process, two ceramic capacitors are serially connected (Fig. 3e). Since the capacitance *C*_L_ of TENG would be furtherly smaller to *C*, when considering a large separation distance, thus we can assume that *Q’* equals 0. In this case, the entire charge 2*Q* from TENG would transfer to ceramic capacitors (3*Q* charge quantity for each). When the two electrodes contact again (Fig. 3f), the capacitance of TENG would increase to *C*_M_ (2 *C*), and consequently, the voltage *V* would decrease, leading to the charge transfer from ceramic capacitors to TENG. During this process, two ceramic capacitors are automatically in parallel connection due to the unidirectional property of diode. Therefore, there would be 3*Q* charges feed back to TENG (Fig. 3g) and thus realize self-charge excitation. After several cycles, the charge would reach saturation (Fig. 3h) and the further charge excitation (when *V* *>* *V*_CE_) would create air breakdown effect (Supplementary Figure 5). In order to ensure the stable output, Zener diode is used to release the surplus charges and control the voltage below the critical value (Fig. 3i). It is worth noting that, if considering a specific step (for instance, firstly the two electrodes get separated, and then let out the charge transfer), during the working process, only mechanical work against electric field force in separation process is applied into the system to increase the system energy (self-excitation mechanism from the energy aspect). In addition, because of the existence of air breakdown, negative charges will transfer from the top electrode to the surface of Kapton film, which would cause the opposite charges on two electrodes when compared with the initial state. In this case, the reverse switch should be applied to restart the system after discharging (Supplementary Figure 6b, 13b and Supplementary Movie 3). The detailed process that charge excited by the triboelectric charges on surface of dielectric film for SCE-TENG with 3 SVMC units are illustrated and discussed in Supplementary Figures 13, 14 and Supplementary Note 6, 7.

### Performance of self-charge excitation nanogenerator

**Fig. 4:**
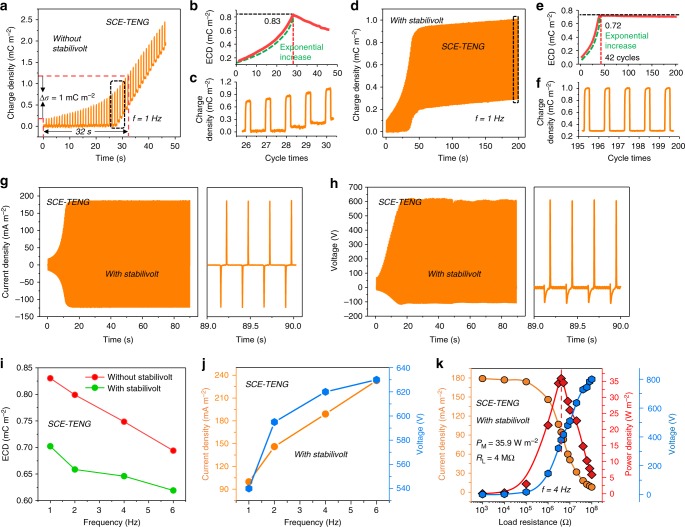
Output performance of self-charge excitation triboelectric nanogenerator. **a** The dynamic output charge process of SCE-TENG without voltage stabilization element under 1 Hz operation frequency. **b** The ECD versus operation cycles. **c** The detailed output charge curve from the dashed area. **d** The dynamic output charge accumulation process of SCE-TENG with voltage stabilization element under 1 Hz operation frequency. **e** The ECD versus operation cycles. **f** The detailed output charge curve from the dashed area. **g**, **h** Dynamic current and voltage output of SCE-TENG with voltage stabilization under 4 Hz operation frequency, respectively, and the right side of each is the enlarged saturated output curve. **i** ECD of SCE-TENG under various operation frequencies with/without voltage stabilization. **j** Current and voltage output of SCE-TENG under various operation frequencies with voltage stabilization. **k** The current, voltage and power output of SCE-TENG with voltage stabilization under various external load (sinusoidal motion with 4 Hz frequency). The thickness of the dielectric Kapton film here is 9 μm

Benefiting from its self-charge excitation through SVMC, the maximum charge density of SCE-TENG up to 1.0 mC m^−2^ can be obtained only in 32 s without voltage stabilization at 1 Hz (Fig. 4a), much faster than that of 201 s for the ECE-TENG (Fig. 2e). It indicates that SCE-TENG offers more effective charge accumulation at lower frequency than ECE-TENG (Fig. 4b). The waveform detail of the charge density from Fig. 4c shows that the baseline starts to shift up rapidly after reaching a critical value. Without voltage stabilization, although the charge density can rapidly reach to 2.5 mC m^−2^ in 46 s (Fig. 4a), the ECD decreases quickly after the maximum. Therefore, an exactly matched Zener diode should be used to realize a stable ECD. Obviously, the charge density with Zener diode is much steadier (Fig. 4d and Supplementary Movie 4), and the ECD needs only 42 cycles to reach the stable state (Fig. 4e) that is much less than that of 300 cycles for the ECE-TENG (Fig. 2j), but the stability is a little worse than that of ECE-TENG. The ECD can reach 0.72 mC m^−2^ and is smaller than that without voltage stabilization since the Zener diode cannot precisely match the critical voltage. Figure 4f shows the waveform detail of the stable charge density with voltage stabilization. The short-circuit current reaches to the stable state quickly in 12 s at 4 Hz, then tends to be long-term stable (Fig. 4g). The output stable current and load voltage are 187 mA m^−2^ (Fig. 4g) and 630 V (Fig. 4h), respectively. It should be noted that the current is a necessary requirement for self-charge excitation in SCE-TENG, thus, we choose the load of 10 MΩ for voltage measurement.

The ECD decreases linearly from 0.83 mC m^−2^ to 0.70 mC m^−2^ without voltage stabilization and decreases from 0.72 mC m^−2^ to 0.62 mC m^−2^ with voltage stabilization in the frequencies of 1–6 Hz as shown in Fig. 4i, indicating that higher ECD can be obtained in longer contact time of TENG (charging/discharge time). Meanwhile, the current and voltage increase with the increase in frequency and reach the maximum of 233 mA m^−2^ and 622 V at 6 Hz, respectively (Fig. 4j). Figure 4k shows the current and output power density at different resistance from 1 KΩ to 100 MΩ, and the maximum power density reaches to 35.9 W m^−2^ with a load of 4 MΩ at 4 Hz, which is a little smaller than 38.2 W m^−2^ obtained by the ECE-TENG.

According to the results above and Supplementary Figure 10b and 11a, the performance could be further improved by proper selection of dielectric material and reduction of the thickness of dielectric layer. In Supplementary Figure 88e-h, the output charge of SCE-TENG with 5-μm dielectric Kapton film are presented, and the effective charge output density can also reach 1.25 mC m^−2^. Comparing the effective charge density curve of ECE-TENG and SCE-TENG in Fig. 2, Fig. 4, Table 1 and Supplementary Figure 8, the charge accumulation of SCE-TENG shows an exponential increase (~30 working cycles to saturation), while ECE-TENG shows a linear increase (~300 working cycles to saturation), which indicates that our proposed SCE-TENG system has a high charge excitation efficiency. In addition, we make a systematical comparison among recent developed charge excitation TENG works in Supplementary Table 2, which clearly demonstrates the advantages of charge excitation strategy proposed in this work.

### Demonstrations of charge excitation nanogenerator

**Fig. 5:**
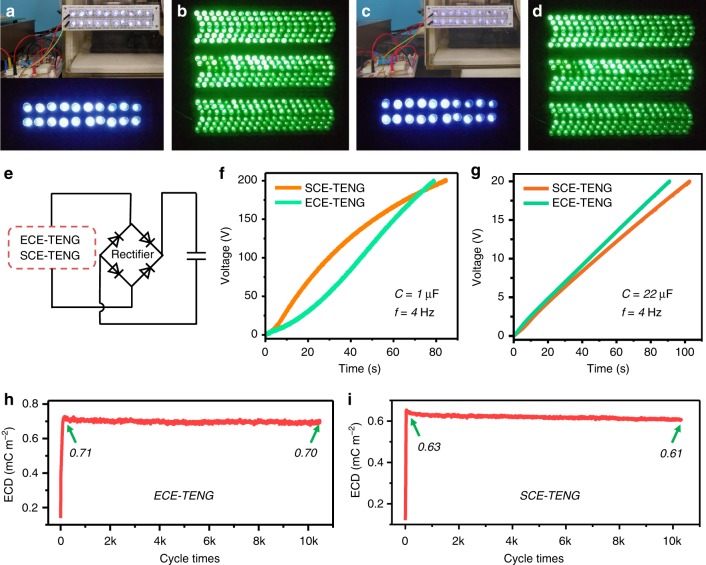
Application of charge excitation nanogenerator to drive devices. **a** The external charge excitation triboelectric nanogenerator (ECE-TENG) lights up 20 white LEDS with diameter of 10 mm in bright and dark environments, and **b** 340 green LEDS with diameter of 5 mm in dark environment. **c** The self-charge excitation triboelectric nanogenerator (SCE-TENG) lights up 20 white LEDS with diameter of 10 mm in bright and dark environments, and **d** 340 green LEDS with diameter of 5 mm in dark environment. **e** The circuit of charging the capacitors. **f** Charging curves of 1 μF capacitor with ECE-TENG and SCE-TENG. **g** Charging curves of 22 μF capacitor with ECE-TENG and SCE-TENG. Effective charge density of **h** ECE-TENG and **i** SCE-TENG with 10,000 operation cycles

To demonstrate the high-output performance of excitation TENG, the main TENG with area 10 cm^−2^ is used to drive various electronic devices and energy storage units at 4 Hz. For the ECE-TENG, 20 white LEDs with diameter of 10 mm in series, and 340 green LEDs with diameter of 5 mm (the power consumption of one LED unit is presented in Supplementary Figure 15) in series (Fig. 5a, b and Supplementary Movie 5) are lighted up effectively in bright and dark environments. Similarly, the identical applications of SCE-TENG are shown in Fig. 5c, d and Supplementary Movie 5. With the help of a full-wave rectifier (Fig. 5e), the ECE-TENG and SCE-TENG can be used to charge a 1-μF capacitor to 200 V in 78 s and 84 s, respectively (Fig. 5f and Supplementary Movie 6), and a 22-μF capacitor to 20 V in 91 s and 102 s with average charging current of 4.8 μA and 4.3 μA, respectively (Fig. 5g). The ECE-TENG has a slightly faster speed than that of SCE-TENG for charging a 22-μF capacitor. The applications above can strongly prove the high-output performance of the charge excitation TENG. Although here we only display the power supply to small electric devices with the small size excitation TENG, we could generate large output energy in large-scaled excitation TENGs.

In addition, stability is also an important factor for TENG. Here, we carried out the stability tests of the ECD for the ECE-TENG and SCE-TENG as illustrated in Fig. 5h, i and Supplementary Figure 16 a, b, from which we can clearly see the quite stable state after 10,000 cycles for the ECE-TENG and SCE-TENG. The shifting of the charge density baseline in Supplementary Fig. 16a, b is due to the charge leakage of the system. The leaked charge quantity of 2 μC and 1.8 μC in 2500 s can be derived from the data for ECE-TENG and SEC-TENG, respectively, which match well with the 1 nA leakage current in Supplementary Table 1.

## Discussion

In this work, we have proposed and developed both an external- and self-charge excitation TENG system in analogy to traditional large-scale magnetic excitation power generation systems. The creative design of VMC and SVMC, with a voltage stabilization component of Zener diode in the ECE-TENG and SCE-TENG, respectively, can achieve high excitation voltage to efficiently supply charges to the electrodes, keep stable output to avoid dielectric breakdown and tune the output power to a desired value. The high ECD of 1.25 mC m^−2^ is obtained by both ECE-TENG and SCE-TENG, and the performance could be further improved by choosing more suitable materials and reducing the thickness of the dielectric layer. This work provides a new platform for TENGs to achieve high and stable power generation by the charge excitation modes for large-scale power applications.

## Methods

### Fabrication of external charge excitation nanogenerator

In order to facilitate the qualitative measurement of the performance of ECE-TENG by linear motor, the excitation TENG and the main TENG were on the same acrylic substrates, which were cut by laser cutter with dimensions of 68 × 45 × 4 mm. Stator: a 68 × 45 × 3 mm liquid cushion was adhered to the bottom acrylic substrates. The liquid cushion was made of 1-mm thick silicone plate and PEG-200 (Liquid) with dimensions of 62 × 40 × 1 mm. A 68 × 45 × 2 mm, 30Psi foam was adhered to the top of liquid cushion. Then, a chamfered 5 mm, 32 × 16.3 × 20 μm Cu electrode for the excitation TENG was adhered to the left side of the upper surface of the foam. For the main TENG, a 42 × 39 × 0.5 mm silicone layer (Ecoflex 10) was adhered to the left side of the upper surface of the foam by mixing the base and the curing agent in 1:1 weight ratio, then cured at room temperature for at least 4 h; a chamfered 5 mm, 32 × 32 × 20 μm Al electrode was adhered to the silicone layer; a 42 × 39 × 9 μm kapton film was attached to the upper surface of Al electrode at last. Oscillator: a 32 × 16.3 × 20 μm Al electrode; and a 42 × 22.3 × 50 μm PTFE film for the excitation TENG was adhered to the left side of the lower surface of acrylic substrate with the PTFE film adhered to the lower surface of Al electrode. A 32 × 32 × 20 μm Al electrode of the main TENG was adhered to the right side of the lower surface of acrylic substrate. For the VMC, the maximum working voltage of rectifier is 1 kV; the capacitance of 6.8 nF for C0-C2 and C6 and 2.2 nF for C3-C5.

### Fabrication of self-charge excitation nanogenerator

The energy for the charge excitation for the SCE-TENG is extracted from the main TENG by SVMC circuit. For the SVMC, the maximum working voltage of rectifier is 1 kV and the capacitance of all capacitors is 10 nF.

### Measurement

Measurement was carried out in a 50 × 50 × 95 cm acrylic glove box. The contact-separation process of TENG was driven by a linear motor (WEINERMOTOR WMU-090-D) with sinusoidal motion in the acrylic glove box. The humidity was controlled by the silica gel desiccant, which was dried by a freeze dryer (Bilon FD-1B-80) for 24 h and a humidifier. The temperature was controlled by a constant temperature circulating water tank (HX-105) and homemade copper tubes with a blower.

The temperature and humidity were measured by a digital temperature-humidity atmospheric pressure gauge (Testo 622). The short-circuit charge, short-circuit current, and voltage of capacitor were measured by an electrometer (Keithley 6514). The load voltage at 10 MΩ of SCE-TENG measured by 6514 with series resistance voltage division method. The open-circuit voltage of ECE-TENG was measured by a 7–1/2 digit graphical sampling multimeter (Keithley DMM7510). The leakage current was measured by the 6514 and a high voltage source (DW N503) provides high voltage. The thickness and surface microscopic appearance of the Kapton was measured by scanning electron microscopy (SEM, TESCAN VEGA 3 SBH SEM).

## Supplementary information

Supplementary Information

Supplementary Movie 1

Supplementary Movie 2

Supplementary Movie 3

Supplementary Movie 4

Supplementary Movie 5

Supplementary Movie 6

Description of Additional Supplementary Files

## Data Availability

The data that support the plots within this paper and other findings of this study are available from the corresponding authors upon reasonable request.

## References

[1] Markvicka EJ, Bartlett MD, Huang X, Majidi C (2018). An autonomously electrically self-healing liquid metal-elastomer composite for robust soft-matter robotics and electronics. Nat. Mater..

[2] Lv T, Liu M, Zhu D, Gan L, Chen T (2018). Nanocarbon-based materials for flexible all-solid-state supercapacitors. Adv. Mater..

[3] Lu C (2018). A stretchable, flexible triboelectric nanogenerator for self-powered real-time motion monitoring. Adv. Mater. Technol..

[4] Zhang L, Dou SX, Liu HK, Huang Y, Hu X (2016). Symmetric electrodes for electrochemical energy-storage devices. Adv. Sci..

[5] Lee Y, Cha SH, Kim YW, Choi D, Sun JY (2018). Transparent and attachable ionic communicators based on self-cleanable triboelectric nanogenerators. Nat. Commun..

[6] Zhu, S., Liu, C., Wang, K., Hu, Y. & Ning, Y. Theoretical and experimental analyses of a hybrid excitation synchronous generator with integrated brushless excitation. *IET Electr. Power App.* **10**, 258–267 (2016).

[7] Fan FR, Tian ZQ, Wang ZL (2012). Flexible triboelectric generator!. Nano Energy.

[8] Zhang C, Tang W, Han C, Fan F, Wang ZL (2014). Theoretical comparison, equivalent transformation, and conjunction operations of electromagnetic induction generator and triboelectric nanogenerator for harvesting mechanical energy. Adv. Mater..

[9] Zhu G, Chen J, Zhang T, Jing Q, Wang ZL (2014). Radial-arrayed rotary electrification for high performance triboelectric generator. Nat. Commun..

[10] Lee KY, Gupta MK, Kim SW (2015). Transparent flexible stretchable piezoelectric and triboelectric nanogenerators for powering portable electronics. Nano Energy.

[11] Zhu G, Peng B, Chen J, Jing Q, Wang ZL (2015). Triboelectric nanogenerators as a new energy technology: from fundamentals, devices, to applications. Nano Energy.

[12] Zi Y (2015). Standards and figure-of-merits for quantifying the performance of triboelectric nanogenerators. Nat. Commun..

[13] Wang J (2016). Sustainably powering wearable electronics solely by biomechanical energy. Nat. Commun..

[14] Chandrasekhar A, Alluri NR, Vivekananthan V, Purusothaman Y, Kim SJ (2017). A sustainable freestanding biomechanical energy harvesting smart backpack as a portable-wearable power source. J. Mater. Chem. C.

[15] Zi Y (2016). Harvesting low-frequency (<5 Hz) irregular mechanical energy: a possible killer application of triboelectric nanogenerator. ACS Nano.

[16] Guo H (2016). A water-proof triboelectric-electromagnetic hybrid generator for energy harvesting in harsh environments. Adv. Energy Mater..

[17] Yi F (2016). A highly shape-adaptive, stretchable design based on conductive liquid for energy harvesting and self-powered biomechanical monitoring. Sci. Adv..

[18] Wang ZL (2017). New wave power. Nature.

[19] Wu C (2017). A spring-based resonance coupling for hugely enhancing the performance of triboelectric nanogenerators for harvesting low-frequency vibration energy. Nano Energy.

[20] Zhang KW, Wang X, Yang Y, Wang ZL (2015). Hybridized electromagnetic-triboelectric nanogenerator for scavenging biomechanical energy for sustainably powering wearable electronics. ACS Nano.

[21] Zhang BB (2016). Rotating-disk-based hybridized electromagnetic-triboelectric nanogenerator for sustainably powering wireless traffic volume sensors. ACS Nano.

[22] Chen J (2017). A fully-packaged and robust hybridized generator for harvesting vertical rotation energy in broad frequency band and building up self-powered wireless systems. Nano Energy.

[23] Wang S, Lin L, Wang ZL (2015). Triboelectric nanogenerators as self-powered active sensors. Nano Energy.

[24] Wang ZL, Chen J, Lin L (2015). Progress in triboelectric nanogenerators as a new energy technology and self-powered sensors. Energy Environ. Sci..

[25] Wang ZL (2017). On maxwell’s displacement current for energy and sensors: the origin of nanogenerators. Mater. Today.

[26] Wei XY, Zhu G, Wang ZL (2014). Surface-charge engineering for high-performance triboelectric nanogenerator based on identical electrification materials. Nano Energy.

[27] Wang S (2016). Molecular surface functionalization to enhance the power output of triboelectric nanogenerators. J. Mater. Chem. A.

[28] Tang W (2015). Liquid-metal electrode for high-performance triboelectric nanogenerator at an instantaneous energy conversion efficiency of 70.6%. Adv. Funct. Mater..

[29] Wang SH (2014). Maximum surface charge density for triboelectric nanogenerators achieved by ionized-air injection: methodology and theoretical understanding. Adv. Mater..

[30] Wang Z, Cheng L, Zheng Y, Qin Y, Wang ZL (2014). Enhancing the performance of triboelectric nanogenerator through prior-charge injection and its application on self-powered anticorrosion. Nano Energy.

[31] Chun J (2016). Boosted output performance of triboelectric nanogenerator via electric double layer effect. Nat. Commun..

[32] Wang J (2017). Achieving ultrahigh triboelectric charge density for efficient energy harvesting. Nat. Commun..

[33] Cheng L, Xu Q, Zheng Y, Jia X, Qin Y (2018). A self-improving triboelectric nanogenerator with improved charge density and increased charge accumulation speed. Nat. Commun..

[34] Xu L, Bu TZ, Yang XD, Zhang C, Wang ZL (2018). Ultrahigh charge density realized by charge pumping at ambient conditions for triboelectric nanogenerators. Nano Energy.

[35] Schaefer, R.C. & Kim, K. Excitation control of the synchronous generator. *IEEE IND. APPL. MAG.* **2**, 37–43 (2001).

[36] Elder JM, Boys JT, Woodward JL (1983). The process of self excitation in induction generators. IEE P-B Elect. Pow. Appl..

[37] Everhart E, Lorrain P (1954). The cockcroft-walton voltage multiplying circuit. Rev. Sci. Instrum..

[38] Cornforth A, Jacob L (1963). Paschen law and shock-excited breakdown in air. J. Appl. Phys..

[39] Lisovskii VA, Yakovin SD (2000). A modified paschen law for the initiation of a DC glow discharge in inert gases. Tech. Phys..

