# Supplementary material for: Integrated charge excitation triboelectric nanogenerator
Source: Nat Commun. 2019 Mar 29;10:1426. doi: 10.1038/s41467-019-09464-8 (PMC6440990; doi:10.1038/s41467-019-09464-8)
Supplement: Supplementary file 1 — Supplementary Information [file 41467_2019_9464_MOESM1_ESM.pdf]

Supplementary Information for

Integrated charge excitation triboelectric nanogenerator

Liu et al.

Supplementary Note 1. The power generation principle of main TENG.

Supplementary Note 2. Theoretical electric performance of main TENG.

Supplementary Note 3. Mechanism of voltage multiplying circuit.

Supplementary Note 4. Critical charge density and opposite charge curve caused by Air breakdown.

Supplementary Note 5. Influence factors on effective charge density

Supplementary Note 6. Working mechanism of SCE-TENG with 3 units SVMC

Supplementary Note 7. Theory of self-charge excitation.

Supplementary Table 1: leak current of different components.

Supplementary Table 2: The systematical comparison of three charge excitation TENG works.

Supplementary Figure 1: Working mode comparison between two external excitation TENGs

Supplementary Figure 2: Simplified working mechanism of main TENG

Supplementary Figure 3: The mechanism of voltage multiplying circuit and ECE-TENG

Supplementary Figure 4: Photographs of wiring circuit board for ECE and SCE TENG

Supplementary Figure 5: Schematic illustration of Air breakdown caused by strong electric field

Supplementary Figure 6: Air breakdown effect on the secondary start of generator

Supplementary Figure 7: Electrical performance of ECE-TENG

Supplementary Figure 8: The charge output performance of ECE and SCE TENG when using 5 um Kapton film as dielectric layer

Supplementary Figure 9: Structures and environments influence on double dielectric layers ECE-TENG

Supplementary Figure 10: Working modes and materials influence on double dielectric layers ECE-TENG

Supplementary Figure 11: Film thicknesses and dielectric layer numbers influence on ECE-TENG

Supplementary Figure 12: Schematic diagram of the circuit evolution of SCE-TENG from ECE-TENG

Supplementary Figure 13: The charge excitation process of SCE-TENG by the charged dielectric films.

Supplementary Figure 14: Equivalent circuit diagram of self-charge excitation process with three VMC units.

Supplementary Figure 15: The power that one LED consumes when lighting 340 green LEDs with diameter of 5 mm in series

Supplementary Figure 16: Ten thousands durability test of the device.

Supplementary Movie 1: Demonstration of the opposite charge curve and secondary start-up for ECE-TENG.

Supplementary Movie 2: Demonstration of charge density curve with voltage stabilization for ECE-TENG.

Supplementary Movie 3: Demonstration of the opposite charge curve and reverse circuit for SCE-TENG.

Supplementary Movie 4: Demonstration of charge density curve with voltage stabilization for SCE-TENG.

Supplementary Movie 5: Demonstration of lighting white and green LEDS for charge excitation TENG.

Supplementary Movie 6: Demonstration of charging 1μF capacitor for charge excitation TENG.

Supplementary Note 1. The power generation principle of main TENG.

The power generation process of the main TENG is shown in Supplementary Figure 2, (I) shows the initial state, C_S is the charge storage ceramic capacitor with a fixed value. A rectifier diode is to prevent charges in TENG flowing back to the voltage source, which is used to excite the charges into the main TENG by the way of providing a high voltage to the main TENG. (II) shows the contact and charging state, where the voltage source provides a voltage to the main TENG and C_S, and under the effect of voltage, charges go to the main TENG. When the main TENG separates again (Ш), the rectifier diode prevents charges flowing back to the voltage source, thus, charge in the main TENG flows to C_S, and there is a current flowing to C_S. It means that the ceramic capacitor is used to store charges of the main TENG. Meanwhile, the voltage of the ceramic capacitor boosts to V_1. When the main TENG contacts again, C_S supplies charges to TENG, and there is a current flowing to TENG shown in (IV). In summary, we achieves charge excitation through applying voltage to the main TENG, and an alternating current is produced by using a fixed value ceramic capacitor to store charges.

Supplementary Note 2. Theoretical electric performance of main TENG.

As shown in Supplementary Figure 2(I), d and ε_r is thickness and relative permittivity of the dielectric film, $x(t)$ is the thickness of air layer, and ε_0 is vacuum dielectric constant. The size of TENG is much larger than the dielectric film thickness d, therefore, the parallel plate capacitor equation (1) and basic electrical relationship equations (2) can be used to get the electric field intensity in air E_{air} and in dielectric film E_D. Q, σ and S is used to represent charge, charge density and area of main TENG.

Parallel plate capacitor equation: $C = \frac{\varepsilon_0 \varepsilon_r}{d} \cdot S$ (1)

Basic electrical relationship equations: $Q = \sigma \cdot S, Q = V \cdot C, V = E \cdot d$ (2)

Inside air gap: $E_{air} = \frac{Q}{S\varepsilon_0}$ (3)

Inside dielectric film: $E_D = \frac{Q}{S\varepsilon_0 \varepsilon_r}$ (4)

And the voltage of main TENG can be given by equation (5):

$$V_M = E_{air} \cdot x(t) + E_D \cdot d = \frac{Qx(t)}{\varepsilon_0 S} + \frac{Qd}{\varepsilon_0 \varepsilon_r S} \qquad (5)$$

According to the equations (2), (5) and $C = Q/V$, we can get the capacitance of main TENG:

$$C_M = \frac{Q}{V_M} = \left(\frac{x(t)}{\varepsilon_0 S} + \frac{d}{\varepsilon_0 \varepsilon_r S}\right)^{-1} = \frac{\varepsilon_0 S}{x(t) + \frac{d}{\varepsilon_r}} \qquad (6)$$

In Supplementary Figure 2(II), an excitation voltage V_E is applied to the main TENG and charge storage capacitor C_S. When the excitation voltage is below its critical value, the relationship of charge Q_{M0} and charge density σ_{M0} in the main TENG with V_E is as follows:

$$Q_{M0} = \frac{\varepsilon_0 \varepsilon_r S}{d} \cdot V_E \qquad , x(t)=0 \qquad (7)$$

$$\sigma_{M0} = \frac{\varepsilon_0 \varepsilon_r}{d} \cdot V_E \qquad , x(t)=0 \qquad (8)$$

We can see that Q_{M0} and σ_{M0} have a complete linear relationship with V_E, it means that the output power of the main TENG can be controlled in/within/at a certain value by adjusting excitation voltage V_E.

Supplementary Figure 2(V) shows a simplified model, where, the main TENG with charge $Q_{M0} - Q_{SC}$ is equivalent to a variable capacitor C_M, which is in parallel with C_S, and there is a short-circuit charge Q_{SC} in C_S. Q_{M0} is a constant when $x(t)=0$, and C_M and C_S have the same voltage. Thus, there is alternating-current when the value of C_M changes into a periodic. According to equation (6), (7) and above, a short-circuit charge Q_{SC} and charge density σ_{SC} equation can be as follows, where V_S is the voltage of C_s.

$$V_M = \frac{Q_{M0} - Q_{SC}}{C_M} = V_E + \frac{Q_{SC}}{C_S} = V_S$$

$$Q_{SC} = \frac{\frac{Q_{M0}}{C_M} - V_E}{\frac{1}{C_S} + \frac{1}{C_M}} = \frac{V_E(C_{M,x(t)=0} - C_M)}{\frac{C_M}{C_S} + 1} \qquad (9)$$

$$\sigma_{SC} = \frac{Q_{SC}}{S} = \frac{V_E(C_{M,x(t)=0} - C_M)}{(\frac{C_M}{C_S} + 1) \cdot S} \qquad (10)$$

According to equation (5), (7), (8), the open-circuit voltage is:

$$V_{OC} = V_M - V_S = \frac{Q_{M0}x(t)}{\varepsilon_0 S} + V_E - V_E$$

$$V_{OC} = \frac{\sigma_{M0}x(t)}{\varepsilon_0} = \frac{\varepsilon_r x(t)}{d} \cdot V_E \qquad (11)$$

In summary, the basic electric output performance (C_M, σ_{M0}, σ_{SC}, V_{OC}) equations of main TENG are shown as follow.

$$C_M = \frac{\varepsilon_0 S}{x(t)+\frac{d}{\varepsilon_r}} \qquad (6)$$

$$\sigma_{M0} = \frac{\varepsilon_0\varepsilon_r}{d} \cdot V_E \quad , \quad x(t)=0 \qquad (8)$$

$$\sigma_{SC} = \frac{Q_{SC}}{S} = \frac{V_E(C_{M,x(t)=0} - C_M)}{(\frac{C_M}{C_S}+1)\cdot S} \qquad (10)$$

$$V_{OC} = \frac{\varepsilon_r x(t)}{d} \cdot V_E \qquad (11)$$

Supplementary Note 3. Mechanism of voltage multiplying circuit.

Supplementary Figure 3b shows the details of VMC, which consists of 7 rectifier diodes and 7 ceramic capacitors and the photograph of the ceramic capacitors and Zener diodes is illustrated in the inset of Supplementary Figure 3a. With an alternating voltage of V_0 input, the output voltage can achieve $6V_0$[1]. Supplementary Figure 3c shows the basic power generation process. First, the oscillator and stator are in separation after initialization (I), where positive and negative charges distribute on the Al electrode and surface of PTFE film of the excitation TENG respectively, and there is no charge in VMC circuit. The open-circuit voltage of excitation TENG is V_0. Second, when the TENG is in contact, the positive charges transfer from Al electrode to Cu electrode and most of the charges go to C6 directly, while a small number of charges go to C0, C1, C2 and the main TENG through rectifier diodes as shown in (II), where the voltage of C6 reaches to V_0 after multiple contacts. Third, when the TENG is in separation in (III), the voltage of C5 reaches to 2 V_0 due to the excitation TENG and C6 in series after multiple separations. Charges in the main

TENG flow to C0, C1 and C2 antd then store in them. Fourth, when TENG contacts again in (IV), the voltage of C2 reaches to 2 V_0 due to the excitation TENG and C5 in series after multiple contacts. Charges stored in C0, C1 and C2 flow to main TENG, and an alternating current forms under contact-separation cycles. Finally, under the charge accumulation effect, the voltage of C0, C1 and C2 in VMC can reach to 2 V_0, and a very high output voltage of 6 V_0 can be achieved to supply the main TENG.

Supplementary Note 4. Critical charge density and opposite charge curve caused by Air breakdown.

As shown in Supplementary Figure 5, a high voltage is applied to the main TENG, a strong electric field is produced due to quite small thickness of the dielectric film, according to the air breakdown by Paschen'law[2], charges transfer from Cu electrode to the surface[3] of dielectric film under strong electric field.

When the charge density is larger than the critical value, air breakdown happens. We call the charge density at air breakdown as critical charge density σ_C.

As figure shows, charges remain on the surface of the dielectric layer after discharging and separating. When the TENG contacts again, there is an opposite current on contact surfaces in Supplementary Figure 5 compared with that in Supplementary Figure 2(IV), therefore, an opposite charge curve appears at the second startup of the main TENG.

Supplementary Note 5. Influence factors on effective charge density

In this section, we study the influence factors on the effective charge density (ECD) with ECE-TENG, and all the measurements are completed at 4Hz, temperature of 293 K, humidity of 5%RH except the humidity research.

The dielectric layer is an important factor in TENG. Therefore, we design the main TENG in ECE-TENG with two identical dielectric layer between the electrodes as shown in Supplementary Figure 9a. The right angles on the rectangle electrode are cut (Supplementary Figure 9b) to avoid the breakdown of the kapton film caused by point discharge under strong electric field. A VMC is

used to rectify current and boost voltage, and a Zener diode is used to stabilize the voltage of main TENG as shown in Supplementary Figure 9a. From Supplementary Figure 9c, we can see that the charge density increases gradually up to 0.8 mC m^{-2} in the main TENG, but the baseline also increases accordingly. Therefore, the effective charge density (ECD) that c is only 0.17 mC m^{-2} (inset in Supplementary Figure 9c). As the strong electric field caused by the charge accumulation applied on the dielectric contact layers can drag some charges from the kapton surface (field emission) and form bond charges on them and causes the useless of some charged electrons on the electrodes, thus the ECD is less than the charge density on the electrodes. With the increase of the charge density on the electrodes, the bond charge density on the dielectric layers increases and causes the baseline increase with the accumulation in charge density. Obviously, there exists a critical charge density (σ_C) on the electrodes and when the charge density exceeds σ_C the field mission happens.

The TENG works in contact-separate mode under periodic reciprocating motion. Therefore, the contact manner directly affects its working efficiency. Four different cushions (three foams and c-cushion) are compared to enhance the ECD as shown in Supplementary Figure 9d, indicating that the composite liquid cushion has the largest ECD of 0.31 mC m^{-2}, photograph in the right shows the actual devices. The influence of humidity on the ECD is shown in Supplementary Figure 9e, from which we can know that the ECD gradually decreases with the increase in humidity from 5%RH to 80%RH for PA66 and kapton. The ECD decreases slowly before 50%RH and has the largest value of 0.36 mC m^{-2} and 0.31 mC m^{-2} at 5%RH for PA66 and kapton, respectively. We also study the influence of different power generation principles on ECD under external-charge-excited mode in Supplementary Figure 10a. There is a small difference of the ECD among the three principles, and we chose principle 1 (Supplementary Figure 9a).

The influence of material and dielectric layer's thickness on ECD is investigated. As shown in Supplementary Figure 10b, PA66 and PP have the best ECD of 0.38 mC m^{-2} under the same thickness, and the inset shows the actual devices of different materials. Limited by actual conditions, we chose the kapton (K) film to study the effect of thickness on ECD. In Supplementary Figure 11a, the ECD gradually decreases with the increase of thickness for the double dielectric layer structure of Al-K / K-Al, from which we can see the higher ECD with thinner K film, and the actual devices of Al-K / K-Al structure are shown in Supplementary Figure 11b. Therefore, a

single dielectric layer structure is chosen to decrease the thickness in our experiment (Fig. 2b). The ECD is effectively enhanced to 0.68 mC m^{-2} with the single dielectric kapton of 9 μm for Al / K-Al structure, and Cu electrode reaches a higher ECD of 0.71 mC m^{-2}. The actual device of single dielectric layer is shown in Supplementary Figure 11c. Supplementary Figure 11d shows SEM images of thickness and surface morphology of the kapton film with thickness of 9 μm, 18 μm, 27 μm and 52 μm, indicating little difference in surface single dielectric structure. Results above confirm that paschen's law is suitable for charge excitation TENG as well [3], and the higher ECD is obtained in thinner thickness[4].

According to the above investigation and actual conditions, we choose composite liquid cushion, temperature of 293 K, humidity of 5%RH, power generation principle 1 and single dielectric layer with 9 μm kapton film and Cu/Al electrodes.

Supplementary Note 6. Working mechanism of SCE-TENG with 3 units SVMC

For the SCE-TENG, the power generation principle of the main TENG is exactly the same as that of ECE-TENG besides the charge storage capacitor C_S, as given in Supplementary Note 1, 2 and Figure 2. Equation (12) and (13) describe C_S with contact and separation states for the SCE-TENG.

$$C_S = \frac{1}{\frac{1}{C0}+\frac{1}{C5}+\frac{1}{\frac{C1C2}{C2+C1}+\frac{C3C4}{C3+C4}}} \quad \text{(separation)} \qquad (12)$$

$$C_S = \frac{1}{\frac{1}{C2}+\frac{1}{C3}} \quad \text{(contact)} \qquad (13)$$

The SVMC consists of 7 rectifier diodes and 6 ceramic capacitors as shown in supplementary Figure 13a. Basic power generation process is illustrated in supplementary Figure 13b. Charges go through different routes in SVMC to achieve self-voltage boosting. In initial contact state (I), the capacitance of main TENG is 2 nF measured by DMM 7510, and each ceramic capacitor in SVMC circuit is 10 nF. The Cu electrode with positive charges is connected to the red endpoint. When the Cu electrode separates from the kapton film, the initial charge Q_I with amount Q in Cu electrode flows to Al electrode through the route shown in (II), where the red arrow indicates the current direction. Consequently, C0 and C5 have charge amount Q, and C1-C4 have a charge amount 0.5Q respectively. There is an inequality charge distribution in the capacitors due to the

special series-parallel structure of capacitors (II). The self-charge excitation happens when the TENG contacts again (III). First, positive charges with amount Q in Al electrode all flows back to Cu electrode through the red route. The release circuit is equivalent to C1 and C4 in parallel, and then they connect C5 and C0 in series, by which the amount Q flows back to Cu electrode for the first time. After that, each residual charge of 0.5Q in C2 and C3 releases in the blue circuit and green circuit as shown in lower part, where C3 charges C0 and C1 through the green route, and C2 charges C4 and C5 through the blue route. Meanwhile, C2 and C3 charges the TENG in series and an excitation charge Q_E of 0.105Q (see Supplementary Note 7 and Figure 14) is charged into the main TENG for the second time. At last, there is a charge amount of 1.105Q in the main TENG at the end of one cycle, demonstrating the achieved self-charge excitation. According to the explanation above, we can see that the total charge Q_T increases on the condition that initial charge Q_I and Q_E must have the same electronegativity in an electrode. It can be described by equation (14). Total charges will gradually approach zero when Q_I and Q_E have opposite electronegativity.

$$Q_T = Q_I + Q_E \qquad (14)$$

With the accumulation of charges, effective charge density no longer increases when the charge density exceeds its critical value σ_C (see Supplementary Note 4). At this moment, charge transfers from Cu electrode to the kapton film as shown in (IV), and most of the transferred charge will remain on surface of the kapton film after discharging and separation. Thanks to these bond charges on the dielectric layer, it is hard for the SCE-TENG to start again after turning off, due to the opposite electronegativity of Q_I and Q_E. To overcome this difficulty, a reverse circuit is made by reconnecting the circuit between main TENG and SVMC as shown in (V) (presented in Supplementary Figure 13bV and Movie 2). Similar to the ECE-TENG, the state of charge and current in SCE-TENG with voltage stabilization are shown in Supplementary Figure 13c.

Supplementary Note 7. Theory of self-charge excitation.

In the circuit as shown in Supplementary Figure 14, C3 and C2 both have a charge of 0.5Q, and there is no charge existing in other capacitors in the initial state. The voltage of each branch is equal under the stable state, therefore, we can get equations as follows:

$$V_M = V_{C3} + V_{C2}$$

$$V_{C3} = V_{C0} + V_{C1}$$

$$V_{C2} = V_{C4} + V_{C5}$$

According to the basic circuit principle, C0 and C1 have the same charge Q_{01} and C4 and C5 have the same charge Q_{45} in the final stable state. In the light of equation above and *V=Q/C*, equations can be got as follows:

$$\frac{Q_E}{C_M} = \frac{0.5Q - Q_E - Q_{01}}{C3} + \frac{0.5Q - Q_E - Q_{45}}{C2}$$

$$\frac{0.5Q - Q_E - Q_{01}}{C3} = \frac{Q_{01}}{C0} + \frac{Q_{01}}{C1}$$

$$\frac{0.5Q - Q_E - Q_{45}}{C2} = \frac{Q_{45}}{C4} + \frac{Q_{45}}{C5}$$

We can get the equation (15) of excitation charge Q_E by solving the above equations.

$$Q_E = \frac{\frac{1}{C3} + \frac{1}{C2} - \frac{1}{(\frac{1}{C0} + \frac{1}{C1} + \frac{1}{C3})C3^2} - \frac{1}{(\frac{1}{C2} + \frac{1}{C4} + \frac{1}{C5})C2^2}}{\frac{1}{C_M} + \frac{1}{C3} + \frac{1}{C2} - \frac{1}{(\frac{1}{C0} + \frac{1}{C1} + \frac{1}{C3})C3^2} - \frac{1}{(\frac{1}{C2} + \frac{1}{C4} + \frac{1}{C5})C2^2}} \cdot \frac{Q}{2} \qquad (15)$$

In this work, C0-C5 have the same capacitance 10 nF, C_M has a capacitance 2.0 nF, and C2 and C3 both have charge Q. There is excitation charge of 0.105Q being charged into main TENG as shown in Supplementary Figure 13(Ш).

Supplementary References

1 Everhart, E. & Lorrain, P. The cockcroft-walton voltage multiplying circuit. *Review of Scientific Instruments* **25**, 394-394 (1954).

2 Cornforth, A. & Jacob, L. Paschen law and shock-excited breakdown in air. *Journal of Applied Physics* **34**, 2914-& (1963).

3 Lisovskii, V. A. & Yakovin, S. D. A modified paschen law for the initiation of a DC glow discharge in inert gases. *Technical Physics* **45**, 727-731, (2000).

4 Wang, S. H. *et al.* Maximum Surface Charge Density for Triboelectric Nanogenerators Achieved by Ionized-Air Injection: Methodology and Theoretical Understanding. *Advanced Materials* **26**, 6720-6728, (2014).

Supplementary Table 1 | leakage current of different components

Component	Leakage current	Voltage
Silicone wire	< 1 nA	1 KV
Rectifier diode	≤ 10 nA	1 KV
Ceramic capacitor	< 1 nA	1 KV
Kapton(9 μm)	≤ 1 nA	500 V
Zener Diode (ECE)	90~110 nA	300 V
Zener Diode (SCE)	300~330 nA	450 V

Supplementary Table 2 | The systematical comparison of three charge excitation TENG works

**Charge Excitation TENG**	*Nat. Commun.* **2018** *9*. 3773	*Nano energy* **2018** *49*, 625	This work			
**Types**	External Charge excitation	External Charge excitation	External Charge excitation		Self-Charge excitation	
**Thickness of dielectric film**	63 µm	5 µm	9 µm	5 µm	9 µm	5 µm
**Excitation TENG (Structure/complexity)**	4 layers / complex	3 layers / simple	3 layers / simple		NO Excitation TENG	
**Connecting circuit**	Rectifier	Rectifier	VMC		SVMC	
			Multifunction / Integration			
**Main TENG (Structure/complexity)**	8 layers / Very complex	5 layers / Complex	3 layers / Simple			
**Voltage output (V)**	922	1290	817	1103	622	850
**Short-circuit current output (mA/m2)**	92.1	288	252	426	233	409
**Maximum effective Charge Density**	0.49 mC/m^2	1.02 mC/m^2	0.81 mC/m^2	1.26 mC/m^2	0.83 mC/m^2	1.25 mC/m^2
**Controllable output**	No	No	Yes			
**Charge accumulation speed (Charge Density Increased by 1mC m^{-2} at 1 Hz)**	320 ~ 400s	70 ~ 450 s	201 s	218 s	32 s	23 s
	Linear increase/Very slow	Linear increase /depend on the size of external TENG	Linear increase /Slow		Exponential increase/Very fast	

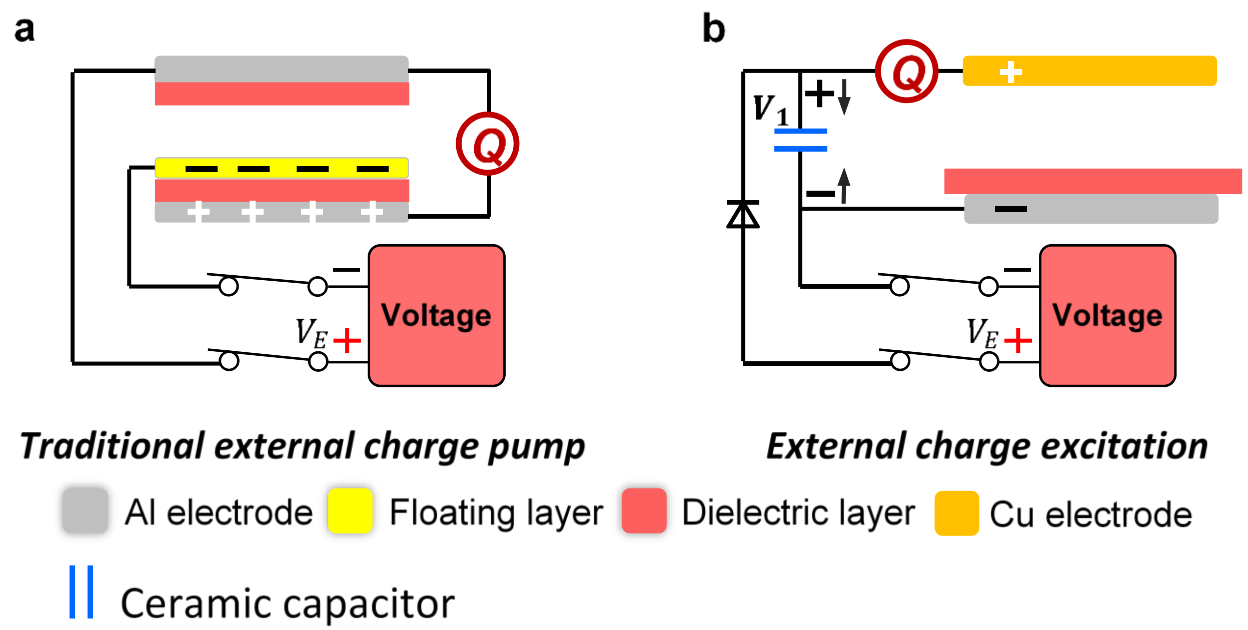

Supplementary Figure 1 | The comparison of basic working mode between traditional external charge pump TENG a and external-charge-excitation TENG b.

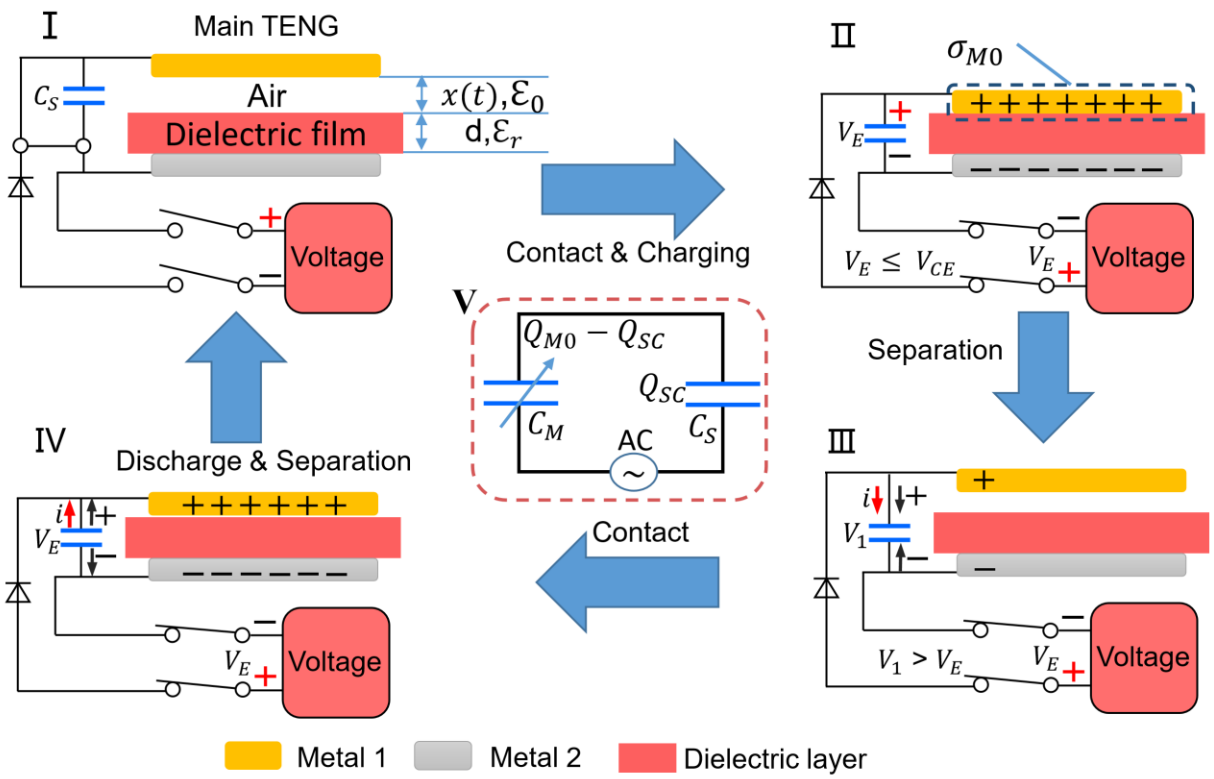

Supplementary Figure 2 | Simplified working mechanism of main TENG.

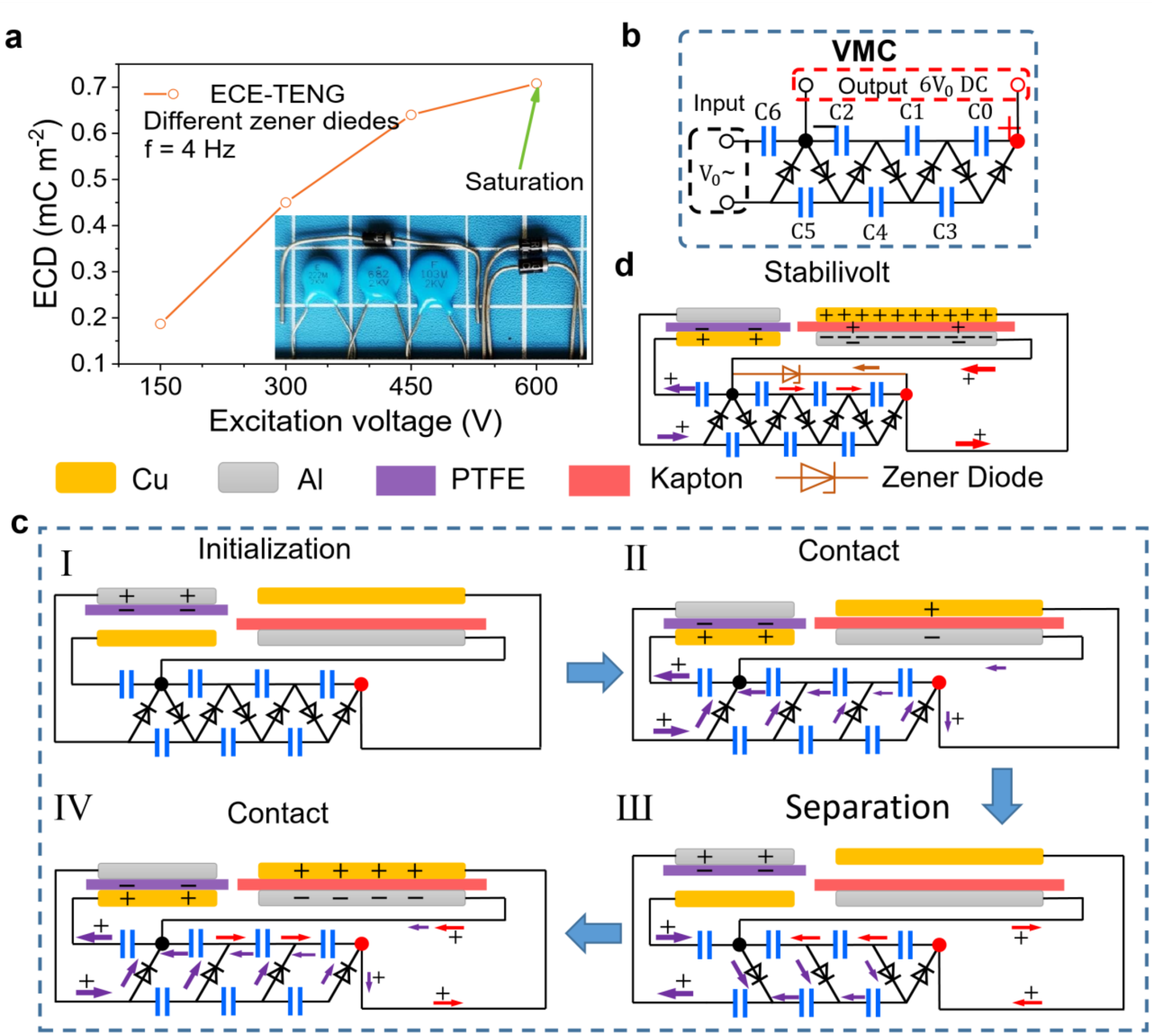

Supplementary Figure 3 | The mechanism of voltage multiplying circuit and ECE-TENG. a, The effective charge density of ECE-TENG under different excitation voltage (which can controlled by the specific Zener diodes). **b,** Electric circuit scheme of the voltage-multiplying circuit. **c,** A complete charge excitation and working cycle of ECE-TENG. **d,** The stable output state of ECE-TENG with voltage stabilization.

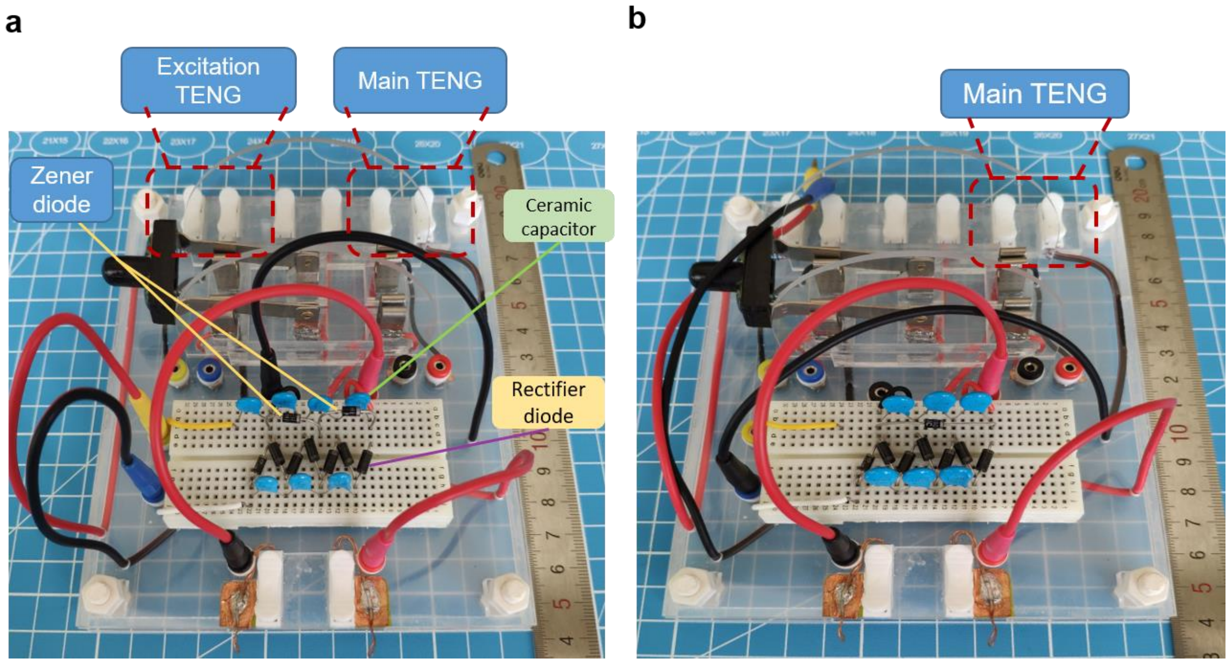

Supplementary Figure 4 | Wiring circuit board which is made to simplify the measurement process for ECE-TENG a and SCE-TENG b.

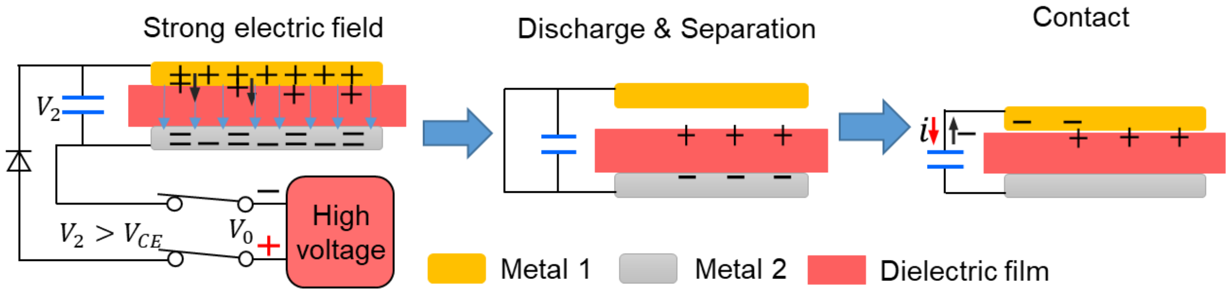

Supplementary Figure 5 | Schematic illustration of Air breakdown caused by strong electric field.

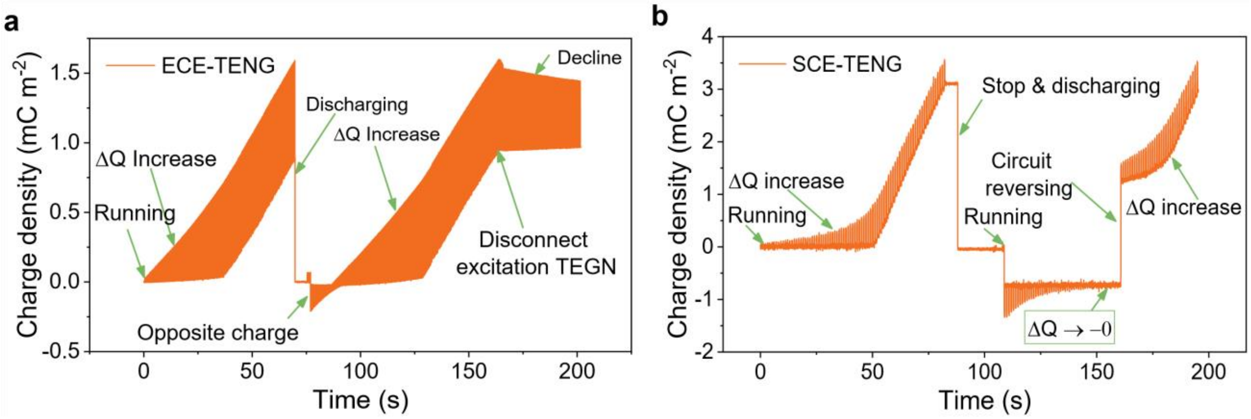

Supplementary Figure 6 | The effect of Air breakdown caused by strong electric field on the secondary start of generator. **a**, The effect of Air breakdown on ECE-TENG. **b**, The effect of Air breakdown on SCE-TENG, and SCE-TENG needs a reverse circuit to start again after discharging.

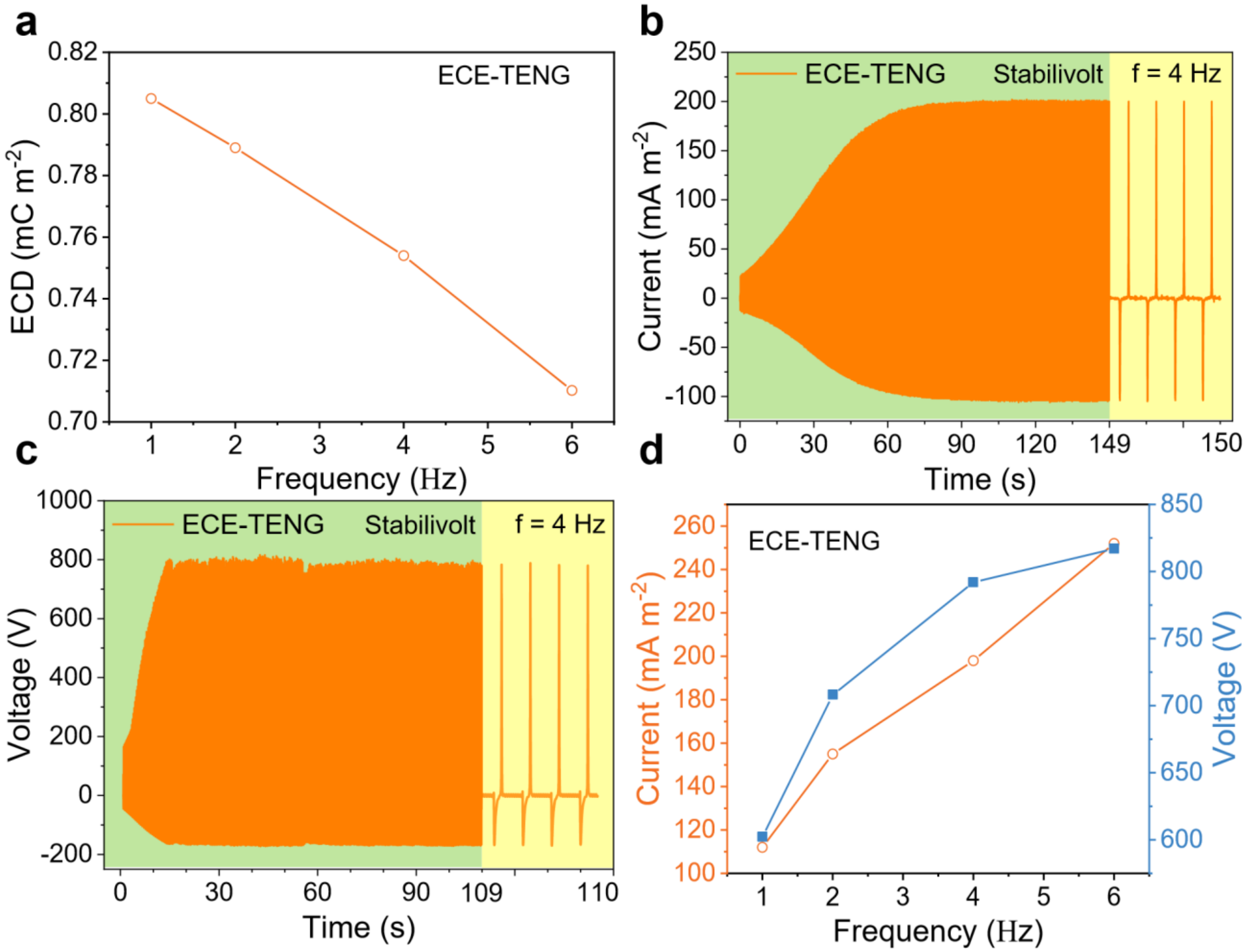

Supplementary Figure 7 | Electrical performance of ECE-TENG. a, Effective charge density from 1 to 6 Hz without voltage stabilization. **b**, Short circuit current with voltage stabilization at 4 Hz, yellow background area is enlarged part at last second. **c**, Voltage with voltage stabilization at 4 Hz, yellow background area is enlarged part at last second. **d**, Short circuit current and voltage from 1 to 6 Hz without voltage stabilization. The thickness of the dielectric kapton film is 9 μm.

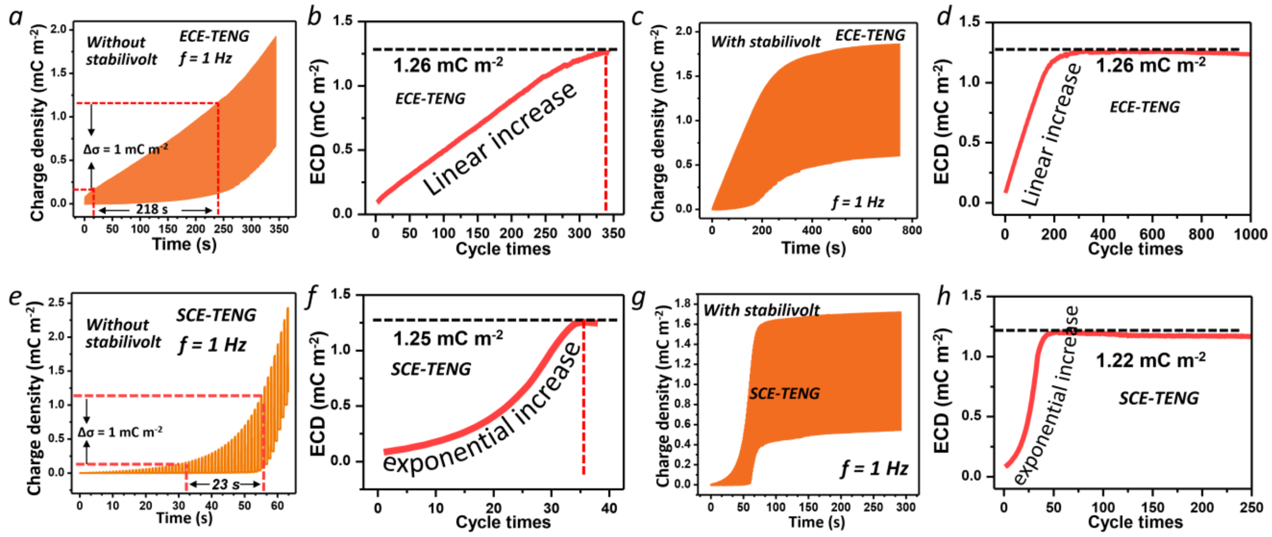

Supplementary Figure 8 | The charge output performance of ECE and SCE TENG when using 5 um Kapton film as dielectric layer.

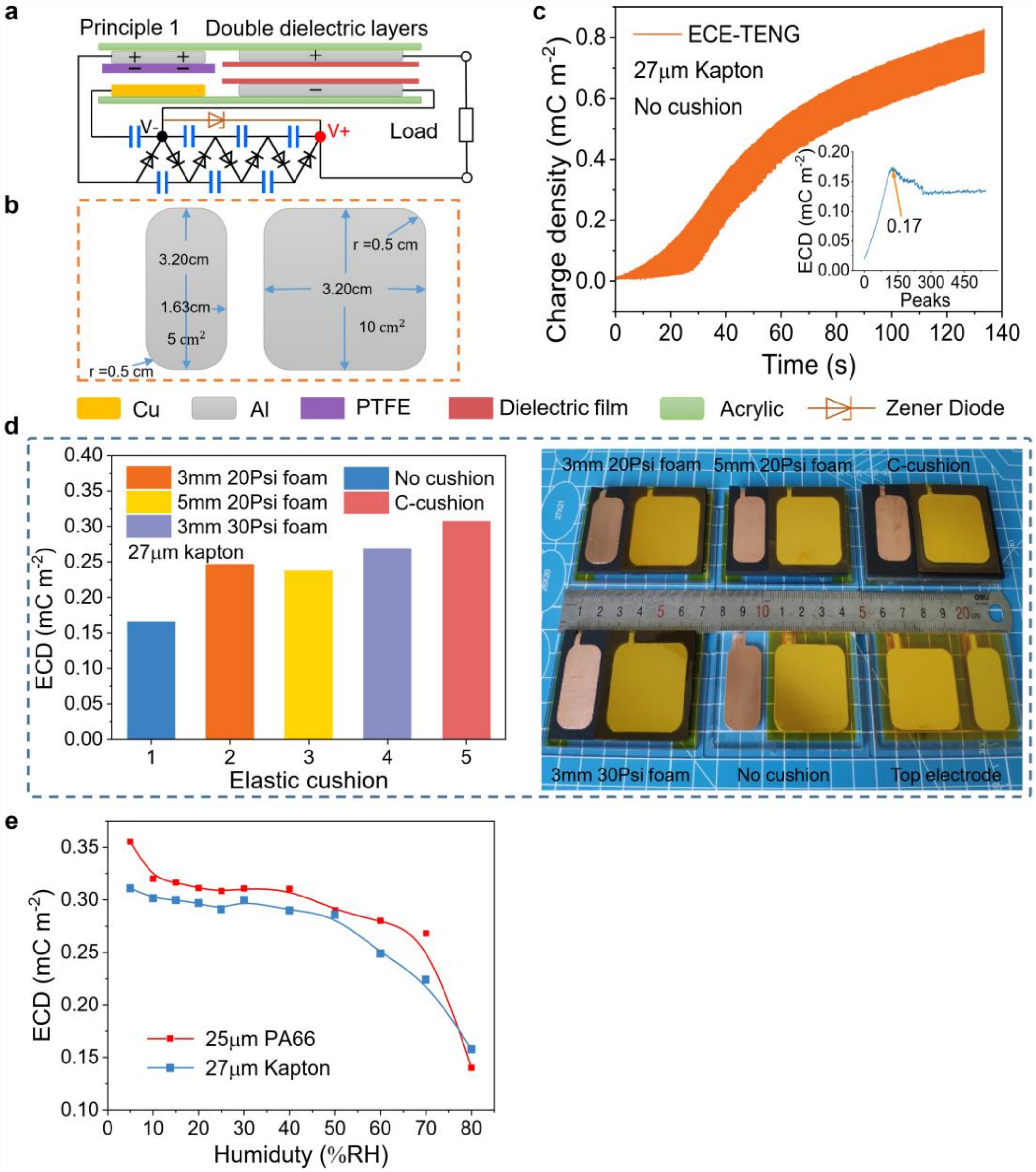

Supplementary Figure 9 | Charge density of double dielectric layers ECE-TENG with different structures and environments. a, Schematic diagram of Double dielectric layers ECE-TENG. **b**, Electrode size of ECE-TENG. **c**, Charge density of double dielectric layers ECE-TENG which has no cushion, insert is effective charge density. **d**, Effective charge density with different elastic cushion structures, c-cushion means composite liquid cushion consists of liquid cushion, foam and silicone layer, photograph on the right is corresponding test devices. **e**, Effective charge density of PA66 and Kapton at humidity from 5% to 80%.

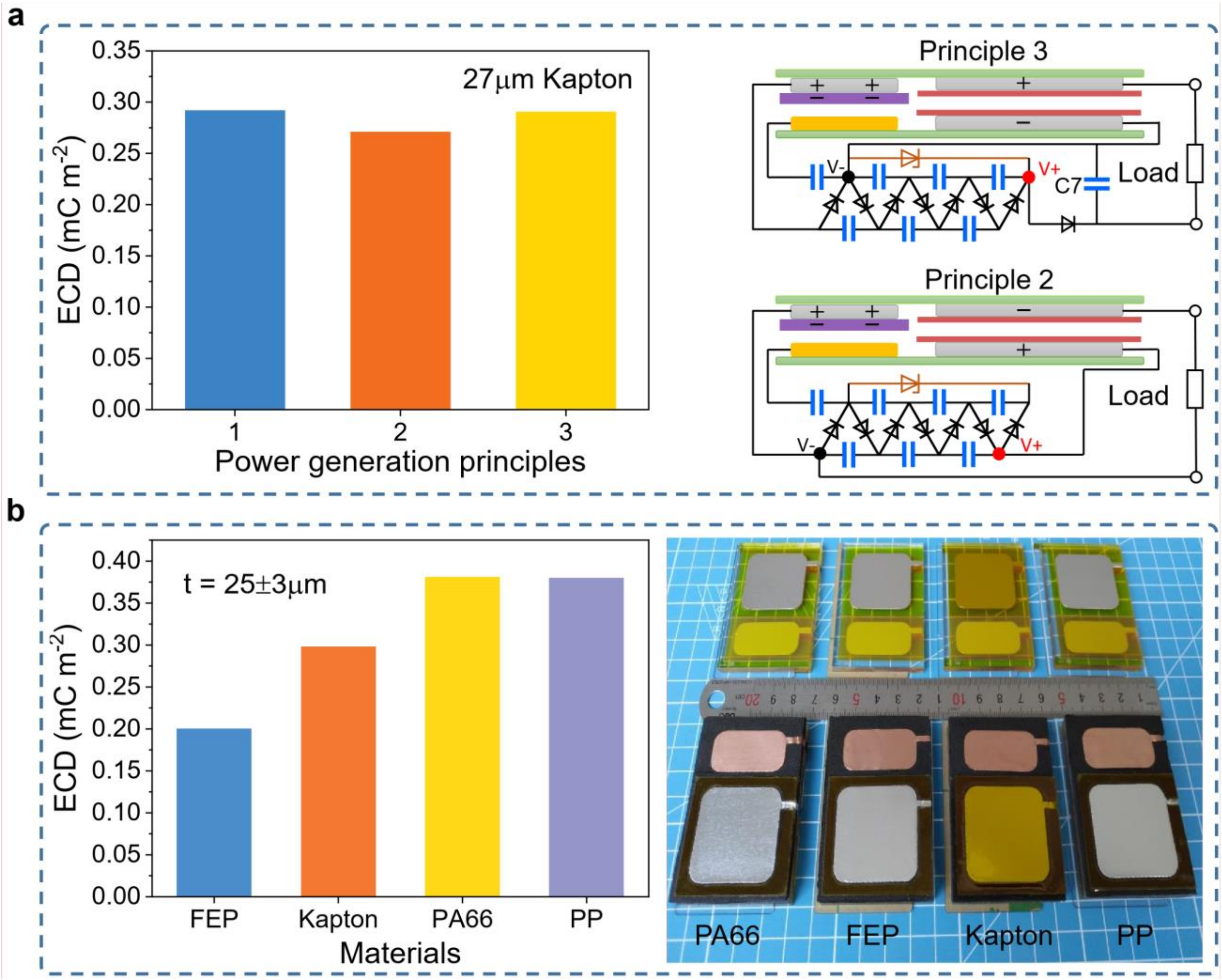

Supplementary Figure 10 | Charge density of double dielectric layers ECE-TENG with different power generation modes and materials. **a**, Effective charge density of three power generation principles . **b,** Effective charge density with different materials, photograph on the right is corresponding test devices.

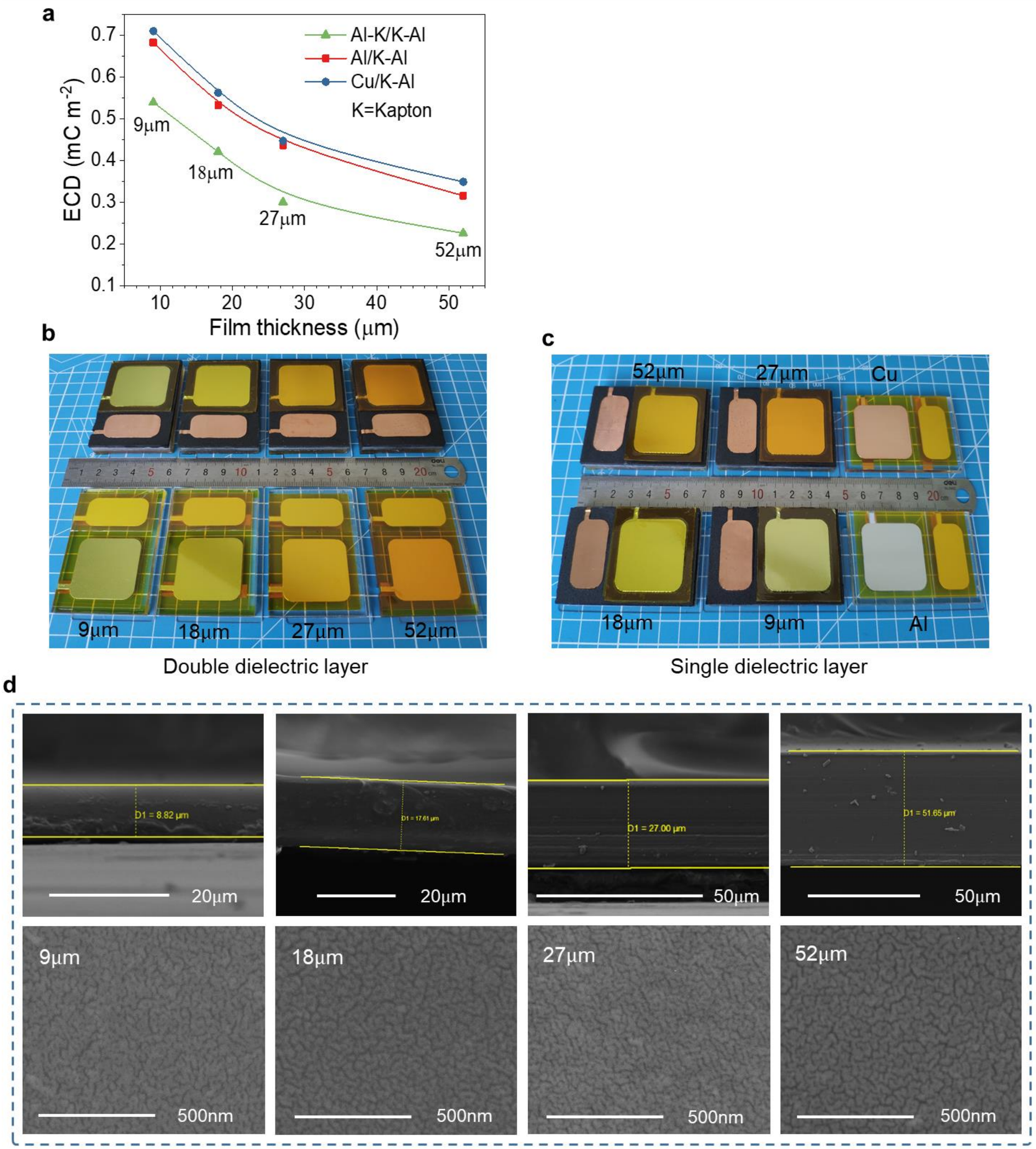

Supplementary Figure 11 | Charge density of ECE-TENG with different film thicknesses and dielectric layer numbers. **a**, Effective charge density of different film thicknesses, single dielectric layer and double dielectric layer. **b**, ECE-TENGs of double dielectric layers with different film thicknesses. **c**, ECE-TENGs of single dielectric layer with different film thicknesses. **d**, SEM images of thickness and surface of kapton film with different thicknesses.

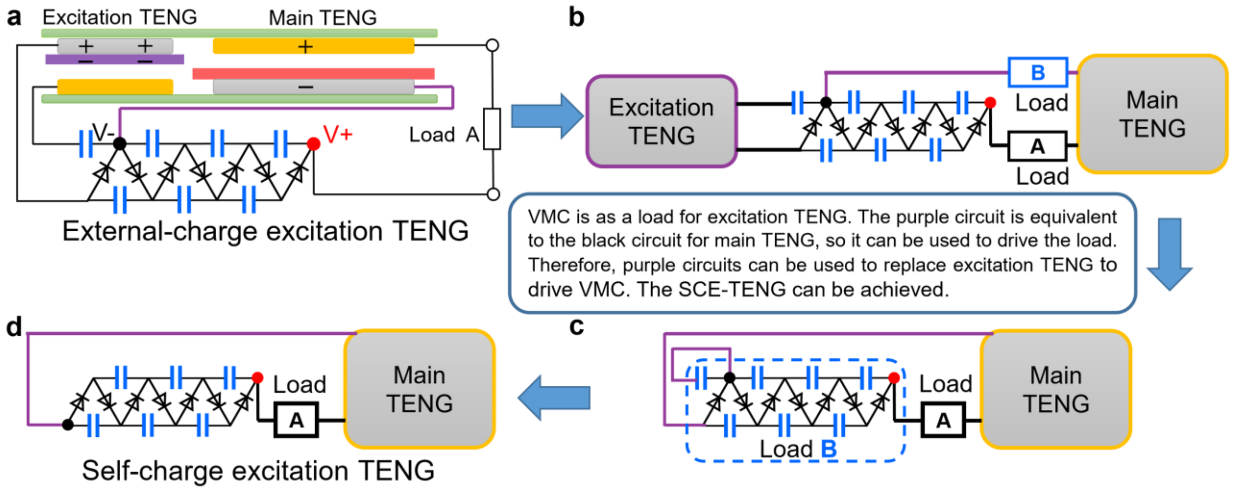

Supplementary Figure 12 | Schematic diagram of the circuit evolution of SCE-TENG from ECE-TENG. a, In the external charge excitation TENG, VMC is as a load for excitation TENG. **b,** As the purple circuit is equivalent to the black circuit for the main TENG, the purple circuit can be used to drive the load. **c,** Therefore, the purple circuit can be used to replace the excitation TENG to drive the VMC as well. **d,** The self-charge excitation TENG can be achieved.

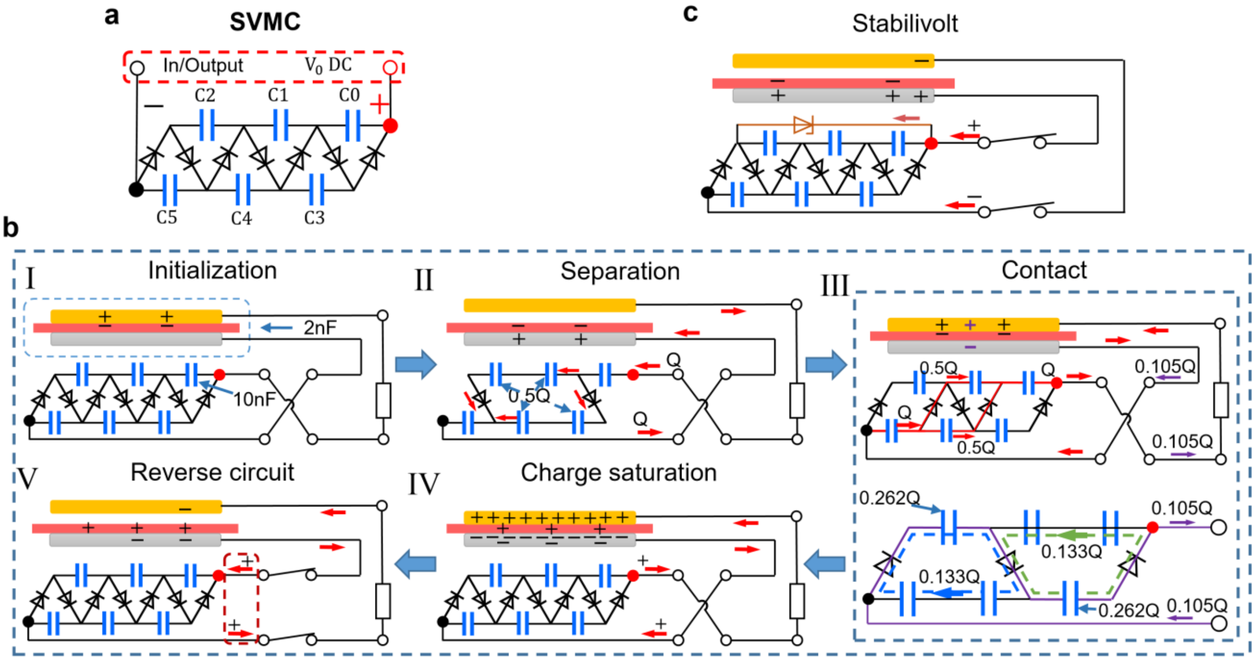

Supplementary Figure 13 | The charge excitation process of SCE-TENG during periodically contact-separation cycle with 3 SVMC units and triboelectric charges on surface of dielectric films. a, Self-voltage-multiplying circuit (SVMC). **b**, Working mechanism of SCE-TENG. **c**, Voltage stabilization state.

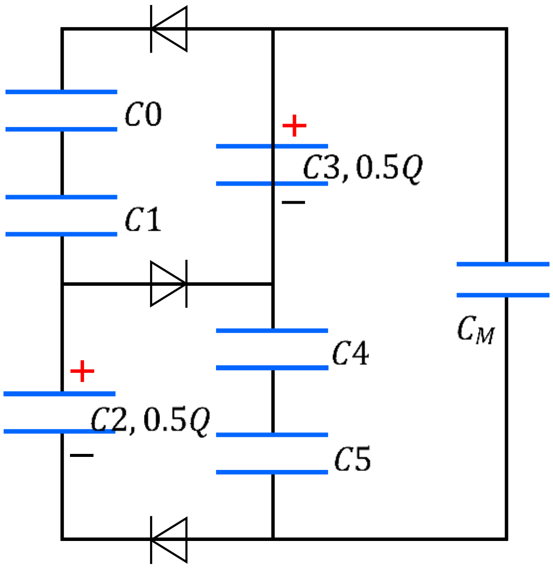

Supplementary Figure 14 | Equivalent circuit diagram of self-charge excitation process with 3 VMC units.

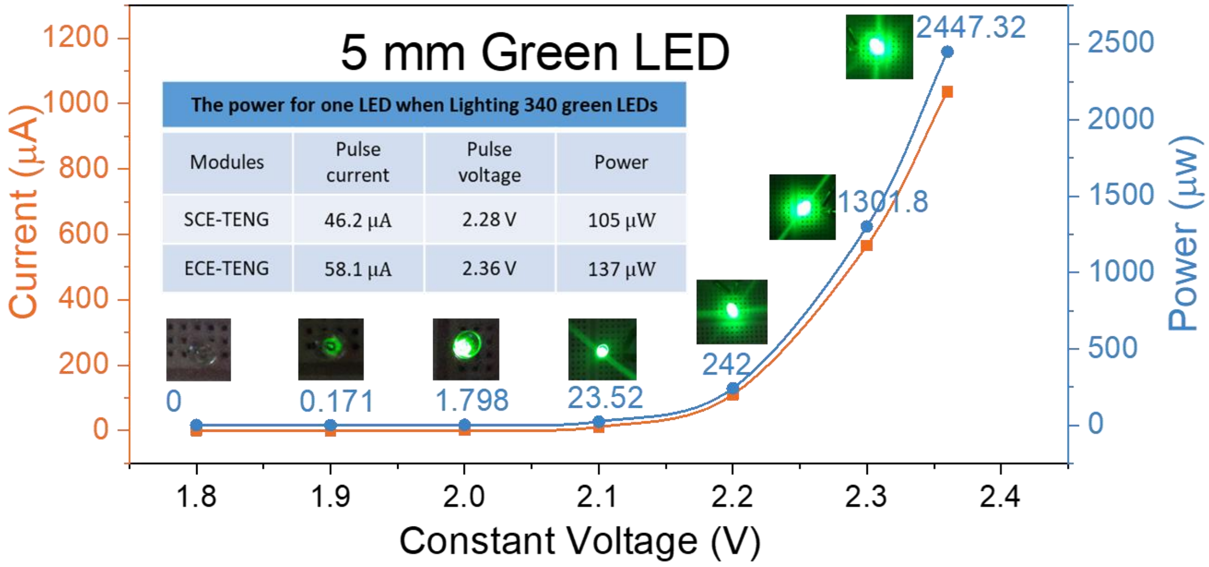

Supplementary Figure 15 | The power that one LED consumes when lighting 340 green LEDs with diameter of 5 mm in series.

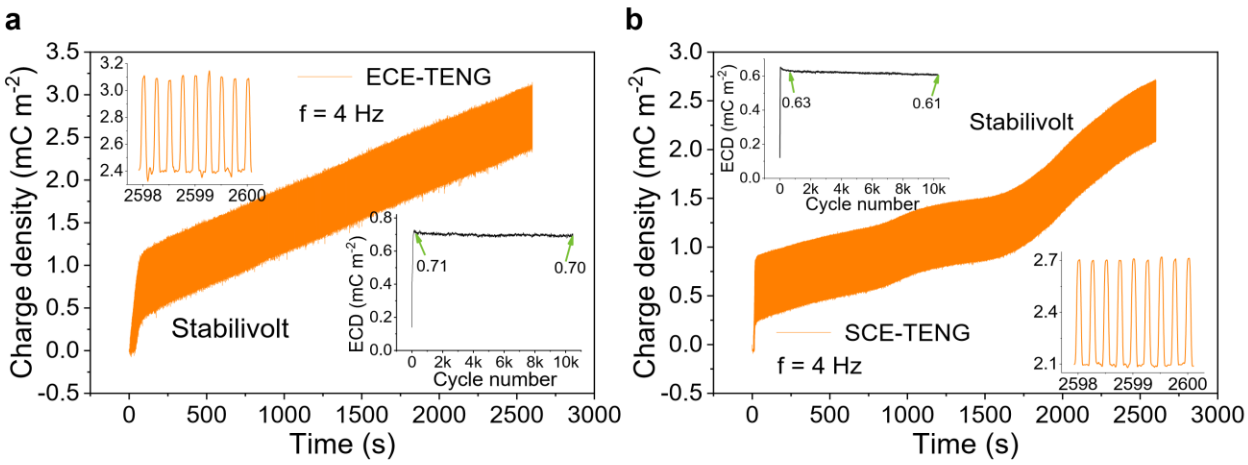

Supplementary Figure 16 | Ten thousand durability test of power system. **a**, Test of ECE-TENG, top and bottom inserts are enlargement at last two second and effective charge density respectively. **b**, Test of SCE-TENG, top and bottom inserts are effective charge density and enlargement at last two second respectively.
