# Supplementary material for: Integrated charge excitation triboelectric nanogenerator
Source: Nat Commun. 2019 Mar 29;10:1426. doi: 10.1038/s41467-019-09464-8 (PMC6440990; doi:10.1038/s41467-019-09464-8)
Supplement: Supplementary file 8 — Description of Additional Supplementary Files [file 41467_2019_9464_MOESM8_ESM.pdf]

Description of Additional Supplementary Files

Supplementary Movie 1: Demonstration of the opposite charge curve and secondary start-up for ECE-TENG.

Supplementary Movie 2: Demonstration of charge density curve with voltage stabilization for ECE-TENG.

Supplementary Movie 3: Demonstration of the opposite charge curve and reverse circuit for SCE-TENG.

Supplementary Movie 4: Demonstration of charge density curve with voltage stabilization for SCE-TENG.

Supplementary Movie 5: Demonstration of lighting white and green LEDS for charge excitation TENG.

Supplementary Movie 6: Demonstration of charging 1μF capacitor for charge excitation TENG.
